# Advancements in mammalian display technology for therapeutic antibody development and beyond: current landscape, challenges, and future prospects

**DOI:** 10.3389/fimmu.2024.1469329

**Published:** 2024-09-24

**Authors:** Peter Slavny, Manjunath Hegde, Achim Doerner, Kothai Parthiban, John McCafferty, Stefan Zielonka, Rene Hoet

**Affiliations:** ^1^ Discovery & Engineering Division, Iontas Ltd./FairJourney Biologics, Cambridge, United Kingdom; ^2^ Technology Division, Iontas/FairJourney Biologics, Cambridge, United Kingdom; ^3^ Antibody Discovery & Protein Engineering, Merck Healthcare KGaA, Darmstadt, Germany; ^4^ Maxion Therapeutics, Cambridge, United Kingdom; ^5^ Department of Medicine, University of Cambridge, Cambridge, United Kingdom; ^6^ Technology Division, FairJourney Biologics, Porto, Portugal

**Keywords:** antibody engineering, antibody libraries, biologics discovery technologies, developability screening, functional screening, genetic engineering

## Abstract

The evolving development landscape of biotherapeutics and their growing complexity from simple antibodies into bi- and multi-specific molecules necessitates sophisticated discovery and engineering platforms. This review focuses on mammalian display technology as a potential solution to the pressing challenges in biotherapeutic development. We provide a comparative analysis with established methodologies, highlighting key aspects of mammalian display technology, including genetic engineering, construction of display libraries, and its pivotal role in hit selection and/or developability engineering. The review delves into the mechanisms underpinning developability-driven selection via mammalian display and their broader implications. Applications beyond antibody discovery are also explored, alongside advancements towards function-first screening technologies, precision genome engineering and AI/ML-enhanced libraries, situating them in the context of mammalian display. Overall, the review provides a comprehensive overview of the current mammalian display technology landscape, underscores the expansive potential of the technology for biotherapeutic development, addresses the critical challenges for the full realisation of this potential, and examines advances in related disciplines that might impact the future application of mammalian display technologies.

## 1 Introduction

From both therapeutic and commercial perspectives, monoclonal antibodies (mAbs) have achieved remarkable success, accounting for six of the ten best-selling drugs in 2023 (1). According to the Antibody Society, over 200 mAb-based therapeutics are approved or under regulatory review by various healthcare authorities [(www.antibodysociety.org/resources/approved-antibodies, date accessed: 4^th^ of May 2024)]. The majority are monospecific mAbs, followed by bispecific antibodies (bsAbs) (2, 3), antibody-drug conjugates (ADCs) (4), and antibody mixtures (5).

Antigen-specific paratopes, the core components of antibody therapeutics, can be generated and identified through various methods. These methods are typically categorised into:

1. *in vivo* methods, such as hybridoma technology and single B-cell technologies (collectively called *in vivo* technologies)

2. *in vitro* methods, such as phage, yeast, ribosome, and mammalian display platform technologies (collectively called *in vitro* display technologies).

**Figure 1:**
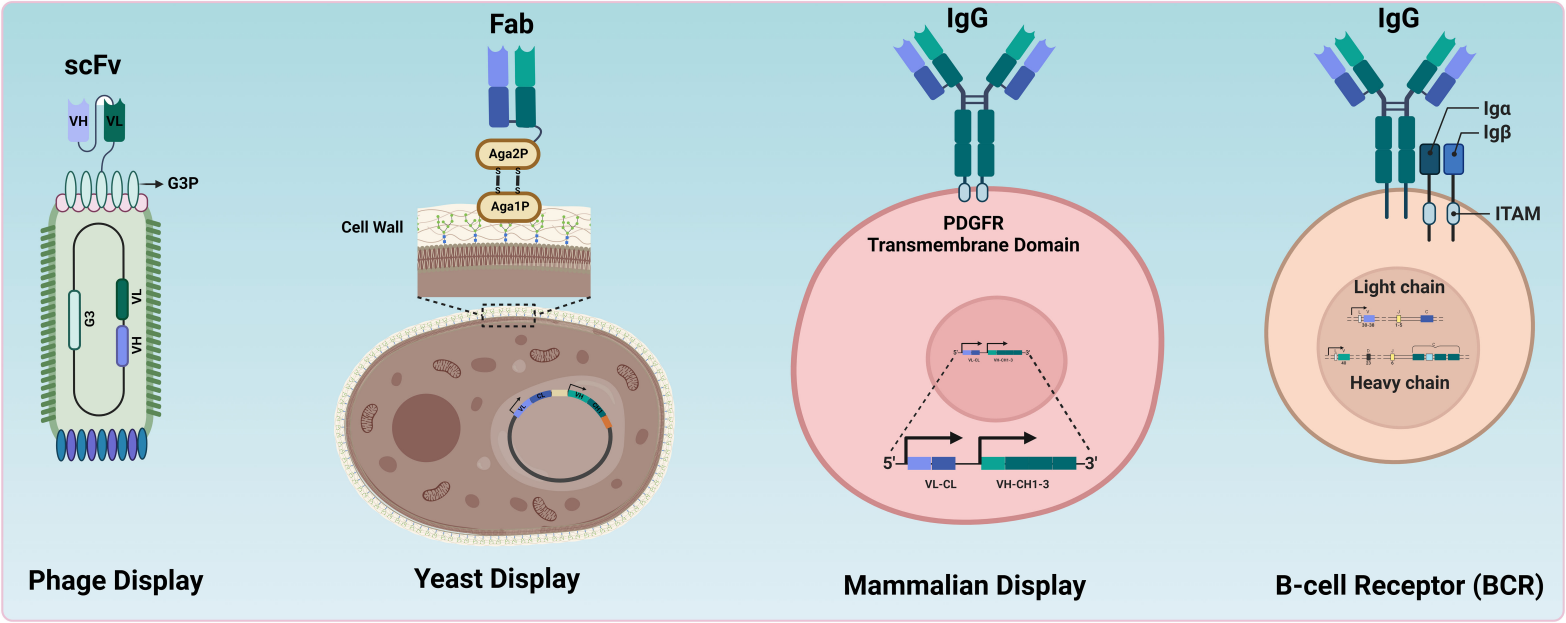
Comparative overview of antibody display systems. Different systems used for antibody or antibody-fragment display. In Phage Display, antibody fragments (e.g., scFv) are expressed on the surface of bacteriophages, enabling the selection of high-affinity binders through panning processes. Yeast Display involves the presentation of whole antibodies or fragments on the cell wall of yeast cells, allowing the selection of binders via fluorescence-activated cell sorting (FACS). Mammalian Display features whole antibodies presented on the surface of mammalian cells, facilitating screening and selection in a more relevant biological context. BCRs illustrate the natural presentation of antibodies on B cells (e.g., memory B cells) for direct screening and selection by FACS.

All these methods physically link the protein of interest to its genetic information, referred to as genotype-phenotype coupling, to enable high-throughput identification of paratopes. The fundamental principles of the most used *in vitro* technologies (phage, yeast display, mammalian display) and *in vivo* technologies (B-cell receptor) displaying antibodies (whole or fragments) are illustrated in **Figure 1**.

### 1.1 *In vivo* technologies

#### 1.1.1 Hybridoma technology

Hybridoma technology developed by Kohler and Milstein in the 1970s (6) is a foundational method for obtaining mAbs. This technology involves immunising animals with a target antigen and fusing antibody-secreting plasma B cells with immortal myeloma cells. The resulting hybrid cells, or hybridomas, are cloned via limiting dilution to obtain stable monoclonal cell lines. Target-specific antibody-secreting clones are then expanded for large-scale antibody production (6). The advantages and limitations of hybridoma technology are summarised in **Table 1A** and reviewed elsewhere (7).

**Table 1A:** Comparison of common *in vivo* antibody technologies.

Technology	Advantages	Disadvantages	References
Hybridoma	• Established and proven technology for generating mAbs for research and therapeutic use • Unlimited supply due to immortal cell line generation • Preserves VH/VL pairing (compared to standard workflows for *in vitro* display technologies) • Applicable to mouse & human transgenic mice • Exploits animal host’s specificity tuning against target, *in vivo* affinity maturation, protein folding and secretion machinery • Compatible with mice immunised with a wide range of target formats (including cDNA/RNA) • Possibility for early IgG Functional Screening compared to *in vitro* display technologies • Scalable antibody production process for screening	• Technology allows mAbs only from limited species (mouse, rat, rabbit) due to limited options for fusion partners • Requires animal immunisation • Low efficiency of fusion of ASCs with myeloma partner to produce hybridomas • Limited mAb diversity (clones) • High costs for culture and maintenance - potential for contamination in cell cultures • Potential genetic drift over time • Requires extensive screening and multiple rounds of plate-based cloning • Rodent cross-reactivity can be challenging • Requires humanisation before therapeutic use	(7–9)
Single B-cell	• Same advantages as Hybridoma Tech. (VH/VL pairing, exploits host’s specificity tuning against target, *in vivo* maturation) • Faster discovery process compared to Hybridoma technology • Potential to screen larger mAb diversity compared to Hybridoma technology and find rare target-specific hits. • Access to a broader pool of immune tissues for B cells • Larger species diversity (including Llama, Human) than hybridoma (immunisation & vaccine/human mAb response) • No (or limited) cell culture needed • Possibility to select against diverse targets • Rapid high throughput selection for target-specificity and function possible via microfluidics-based screening platforms	• High initial setup costs for screening technology and training (FACS, Microfluidics) • Limited supply of short-lived memory and plasma B cells for screening • Challenges in single-cell RT-PCR-based recovery of VH and VL for downstream cloning, expression, and characterisation) • Requires separate screening workflow and technologies for memory and plasma B cells • Markers for phenotypic characterisation and enrichment of desired B cell subtypes (memory or plasma B cells) are limited for certain species • Technically complex assay setup and optimisation needed for high throughput early functional screening	(10–13)

#### 1.1.2 Single B cell technologies

Single B cell technologies have emerged as powerful tools for therapeutic antibody discovery, offering significant advantages over hybridoma technology in speed and efficiency (**Table 1A**). These technologies have been successfully deployed in developing therapeutic antibodies, with several candidates currently under clinical evaluation (14, 15). Advances in high-throughput screening techniques based on Fluorescence-Activated Cell Sorting (FACS) and Microfluidics enable the direct screening of single B cells isolated from various immunised animals, facilitating rapid identification of rare target-specific antibodies. B cells isolated from immune tissues require further processing and enrichment to obtain viable cell sub-populations of interest, such as memory B cells and antibody-secreting cells (ASCs) (plasmablasts and plasma cells) for screening but are often limited in numbers and short-lived. Recent advances in *ex vivo* B cell culture, particularly in the activation and expansion of memory B cells and differentiation into ASCs, have expanded the pool of cells available for single-cell screening and antibody discovery [reviewed elsewhere (10, 11)].

Recent progress in microfluidics-based single-cell screening techniques has transformed antibody discovery from B cells, particularly by enabling high throughput compartmentalisation and interrogation of individual ASCs. ASCs derived from immunised animals secrete a high frequency of target-specific high-affinity antibodies. However, they have limited or no expression of antibodies on the cell surface and cannot be screened using FACS. Microfluidics enables the precise isolation and analysis of single ASCs within microdroplets (16–18) or microchambers (19, 20) to identify rare antibodies of interest. Single ASCs can be screened to identify high-affinity antibodies binding to a soluble or membrane-embedded target of interest (14, 21–23). A few advanced microfluidic platforms can also select antibodies directly based on desired functional attributes (15, 24, 25), further accelerating discovery timelines.

Single B cell technologies leveraging the natural immune antibody repertoire offer several advantages over *in vitro* display technologies. These advantages include natural immune selection for high specificity against the target antigen and effective *in vivo* affinity maturation processes (26, 27) (**Table 1A**). Another critical feature of single B cell technologies is the ability to recover cognate pairing of antibody variable heavy (VH) and variable light (VL) chains from sorted single target-specific B cells (28–30). Retrieval of natively paired VH and VL sequences from the original antibody repertoire produced *in vivo* in response to the target antigen ensures that isolated antibodies retain their natural binding and functional characteristics. This contrasts with most *in vitro* display technologies where the fidelity of natural VH-VL pairing is lost even when B cells from immunised animals are used as starting material due to bulk amplification of VH and VL during library preparation. Several groups have independently developed microdroplet-based microfluidics methods for high throughput recovery of natively-paired VH-VL sequences from single B cells and expressed these paired libraries using phage or yeast display for convenient and iterative screening (31–36). This combination leverages the strengths of both *in vivo* and *in vitro* technologies, enabling the rapid screening and optimisation of antibodies.

### 1.2 *In vitro* display technologies

Antibody display systems such as phage, ribosome, yeast, and mammalian display have been used to identify paratopes with desired properties, leading to marketing approval of multiple mAb therapeutics (37, 38). Major display technologies relying on prokaryotes or lower eukaryotes are compared in **Table 1B** and have been reviewed elsewhere (37, 57, 58). The most frequently used *in vitro* display technologies, phage and yeast display, are summarised below before reviewing mammalian display technologies.

**Table 1B:** Comparison of common *in vitro* antibody display technologies.

Display system	Library size	Format of mAbs	Advantages	Disadvantages	References
Phage	10^10^ - 10^12^	Fab, scFv, VHH, Diabody	• Robust and most widely used display technology • The relative ease of screening, sequencing, and production of soluble protein • Large library size • Compatible with a variety of target formats, including targets expressed on mammalian cells • Cost-effective compared to yeast/mammalian display	• Only displays antibody fragments, not whole IgG • Requires additional screening steps to evaluate full IgGs for functional activity • Potential bias in the library due to phage replication • Longer selection cycles compared to some other methods	(38–42)
Yeast	10^8^- 10^9^	IgG, Fab, scFv, VHH, Bispecific	• Quality control mechanisms of a eukaryotic secretory pathway • Quantitative library screening through magnetic separation combined with FACS • Ability to perform more complex multiparametric selections using FACS • Selection based on expression level	• Smaller library sizes compared to phage and ribosome display • Different glycosylation in yeast compared to mammalian cells makes functional cell assays challenging (e.g., no ADCC or CDC assays) • Transformation efficiency and library complexity limitations • Potential differences in protein folding and stability	(43–47)
Ribosome	10^12^- 10^15^	Fab, scFv, VHH	• Larger libraries compared to phage display • Fast selection system • Completely *in vitro* system, allowing direct manipulation of the selection environment • No requirement for cell transformation	• Sensitive system (RNA) • Mainly restricted to recombinant targets • No screening for developability • High susceptibility to RNase contamination • Challenges in maintaining stable ribosome-mRNA complexes • Lack of post-translational modifications	(48, 49)
Mammalian	10^5^- 10^9^	IgG, Fab, scFv, VHH, Bispecific	• Full-length antibodies and alternative fragment formats (e.g., scFv, Fab, bispecifics) • Appropriate post-translational modifications • Ability to perform more complex multiparametric selections using FACS • Allows earlier functional screening/selection (including those reliant on mammalian glycosylation, e.g., ADCC & CDC) • Sensitive selection for drug-like developability properties • More physiologically relevant system for human therapeutics • Can assess protein folding, stability, and expression in a mammalian context	• Requires tissue culture facilities and related equipment • High initial setup costs for screening technology (e.g., FACS) and training • Limited library size that is dependent on the principle used for library generation • Might need a combination with other display technologies for the selection of non-immune libraries • High costs for culture and maintenance - potential for contamination in cell cultures	(50–56)

#### 1.2.1 Phage display

Phage display technology, invented by George P. Smith in 1985 to display peptides (59), was later adapted in 1990 to display the antibody fragments on phage (60). Since then, the technology has successfully facilitated the discovery of hundreds of antibodies for research, diagnostic, and therapeutic applications, including more than 17 clinically approved antibodies (38, 61). Compared with other display technologies, such as yeast and mammalian display, one of the advantages of phage display is the ability to create large libraries with diversities of up to 10^11^ unique clones. Robust *in vitro* phage selection procedures have been developed, allowing selection on a variety of antigen sources [e.g. protein, peptide, cells, virus-like particles (VLPs), nanodiscs, liposomes – reviewed elsewhere (39)] to identify molecules with specificity to desired epitopes, including the use of sophisticated FACS-based selections on phage to identify rare antibodies against challenging targets (62).

Phage display typically uses filamentous bacteriophage M13 of *E. coli* and can be exploited to express various antibody formats. Antibody fragments are usually fused to the minor coat protein encoded by gene 3 (pIII), present at 3-5 copies per phage. In the early phage systems, the pIII fusion was created in the phage genome (60). To enable the construction of large libraries, a phagemid system is more commonly used, where the Ab-pIII fusion is encoded on a plasmid bearing a phage single-stranded origin of replication (63–65). The assembly of phage particles is then facilitated by co-infection of *E. coli* with helper phage to provide all crucial components for M13 phage packaging. Due to the competition between pIII from the helper phage and the phagemid encoded Ab-pIII fusion, display levels are typically low (e.g., 10% of phage displaying a single copy). To overcome this, a helper phage lacking gene 3 (“Hyperphage”) has been created to increase display levels and enable avidity-based selections (66, 67) [reviewed elsewhere (39, 68)].

Antibody fragments, such as Fragment antigen binding (Fab) (40) or single chain variable fragment (scFv) (42), are commonly used formats in phage display (**Figure 1**, **Table 1B**). Single-domain antibodies (sdAbs) can be displayed and selected using phage display technologies (69–72). Those sdAbs naturally evolve in camelids and cartilaginous fishes as antigen-binding sites of heavy chain-only antibodies (73–75). Camelid-derived VHH (variable domain of the heavy chain of a heavy chain-only antibody) domains have proven versatile for constructing therapeutic modalities (76–78). While phage display is highly effective for selecting specific binders from antibody fragments, one limitation is its inability to display whole antibodies (e.g., IgG), necessitating early transfer to IgG in a mammalian expression system for the selection of antibodies with specific function (e.g., agonism).

#### 1.2.2 Yeast display

Yeast display is another powerful recombinant antibody selection system that uses genetically engineered yeast cells instead of bacteriophages (43). Antibody fragments (e.g., scFvs) were initially displayed on the surface of yeast cells, allowing for the screening and isolation of specific binders (44). The concept behind yeast display involves genetically fusing the gene encoding the protein of interest with a gene encoding a yeast cell wall protein. This fusion gene is then expressed in yeast cells, resulting in the display of the protein within the cell wall. *Saccharomyces cerevisiae* is the most used yeast species for this purpose, being a well-studied and easily manipulated organism.

The most frequently used yeast display system relies on the α-agglutinin complex, composed of subunits Aga1p and Aga2p. Aga1p is integrated into the yeast genome, while Aga2p is plasmid-encoded. Whole antibodies or fragments are genetically fused to either the *N-*terminus or *C-*terminus of Aga2p. This fusion gene is then transformed into yeast cells. Expression of the antibody formats is typically controlled by an inducible promotor, most often the galactose-inducible GAL1/GAL10 promoter system. Once the protein is displayed on the yeast cell wall, it can be screened or selected using various techniques. FACS can isolate yeast cells displaying antibodies with desired specificity (45). Alternatively, magnetic-activated cell sorting (MACS) can be employed by labelling the target protein with magnetic beads and isolating cells displaying these beads (43–45).

Yeast display offers several advantages over phage display, including performing more complex multiparametric selections using flow cytometry (79). In addition, it is claimed that yeast display selects for certain favourable biophysical properties essential for the developability of the final molecules (80–83). However, transformation efficiency and library complexity limit the number of variants displayed on the yeast cell surface (**Table 1B**). It is typically more challenging to perform yeast display selections on complex membrane-embedded targets expressed on mammalian cells [e.g., G protein couped receptors (GPCRs)], and glycosylation patterns of whole antibodies differ due to the glycosylation machinery inside the yeast cells compared to mammalian cells (**Table 1B**). While the scFv architecture is the most frequently used format in yeast display, other antibody fragments may be displayed on the yeast surface, including Fabs, IgGs, and sdAbs (43). Yeast display and phage display technologies have been elegantly combined to leverage enormous library diversities achievable in phage display and the power of FACS with yeast display to select high-quality molecules (84).

### 1.3 Why mammalian display? Increasing requirements in novel therapeutic antibody development

The commercial and clinical requirement for molecules with optimal biophysical properties for manufacture and administration has increased dramatically over the past decade. The term “biophysical properties” in this context refers to the myriad physical and chemical properties of proteins. These can be further divided into categories, for example, properties of the native protein fold (i.e. colloidal properties such as surface charge, hydrophobicity and solubility), or properties relating to the stability of tertiary structure itself (such as thermostability). The impact of each parameter on the suitability of a molecule for development as a drug candidate, and the methods available for interrogating them, is is a matter of intense interest in the field and is discussed extensively elsewhere (85–88). Stable formulation at high concentrations (>100 mg/mL) for subcutaneous administration is increasingly a requirement for commercial competitiveness, particularly in chronic disease settings (89–91). This trend is driven by the importance of patient convenience in a crowded market. The biophysical characteristics that determine the viability of a candidate molecule for development into a marketed drug product, referred to collectively as “developability”, have become a focal point in the early stages of drug development for both simple IgG molecules and more complex modalities such as bi- and multi-specific mAbs. As the final production vehicle for biologics, mammalian cells are uniquely suited to accurately interrogate developability properties and assess other vital molecular features in the final drug format.

The complexity of biologics modalities is rapidly increasing. There is an expanding range of bi- and multi-specific formats in clinical development, often exploiting novel biological functions that depend on finely tuned interactions with multiple targets (for a recent review, see (2, 92)). Furthermore, incorporating non-antibody elements, such as TCRs, cytokines, knotted peptides, and other moieties, into multi-specific biologics has become more common. As of December 2022, approximately 300 bi- or multi-specific antibodies were under investigation in clinical trials (93). These molecules often have intricate architectures involving different paratopes, functionalities and valences (94, 95). The assembly of diverse paratopes or binding components into the final antibody architecture results in an extensive combinatorial space, necessitating the rapid screening of millions of multi-specific combinations to identify the rare optimal candidates. Engineering of such molecules can be laborious, although novel technologies have been developed to address this challenge (96, 97). The task is further complicated by the need to identify developable multi-specific combinations.

Moreover, the biophysical properties of individual binding components are not necessarily predictive of behaviour in the final molecular format of the drug. Sometimes, the optimal multi-specific architecture is not apparent and must be assessed empirically during the discovery and optimisation phases. Thus, including format diversity in combinatorial libraries can be advantageous (e.g. varied linker lengths or relative positioning of binding moieties). Further benefits can be gained in modalities where straightforward functional readouts are possible in a mammalian cell background, and variations in effector domain architecture can be addressed during discovery (e.g., chimeric antigen receptors (CARs) and bispecific CAR applications).

Overall, the rapidly changing modality landscape, particularly the increase in the number and complexity of drug formats, has created a need for more sophisticated biologics engineering platforms. These platforms must address multiple properties of putative drugs simultaneously in their final molecular format of the drug. A platform combining these features with the ability to address developability at a scale appropriate for screening combinatorial libraries would offer significant advantages over traditional biologics discovery and engineering approaches. Mammalian display technology can potentially address some of these critical emergent challenges in biotherapeutic development.

## 2 The basics of mammalian display

Mammalian display technology relies on delivering transgene repertoires into a complex eukaryotic cell background in a manner that simultaneously preserves the genotype-phenotype linkage and achieves an efficiency sufficient for creating large libraries. Once suitable libraries have been constructed, the encoded proteins must be displayed on the cell surface to facilitate the enrichment of clones based on desired molecular properties (their phenotype). This “selection” or enrichment process is fundamental to all display technologies and, in conjunction with the input diversity and the screening experiment design, determines the output quality. Following selection, it is necessary to recover the genes of interest to identify and characterise hits.

**Figure 2:**
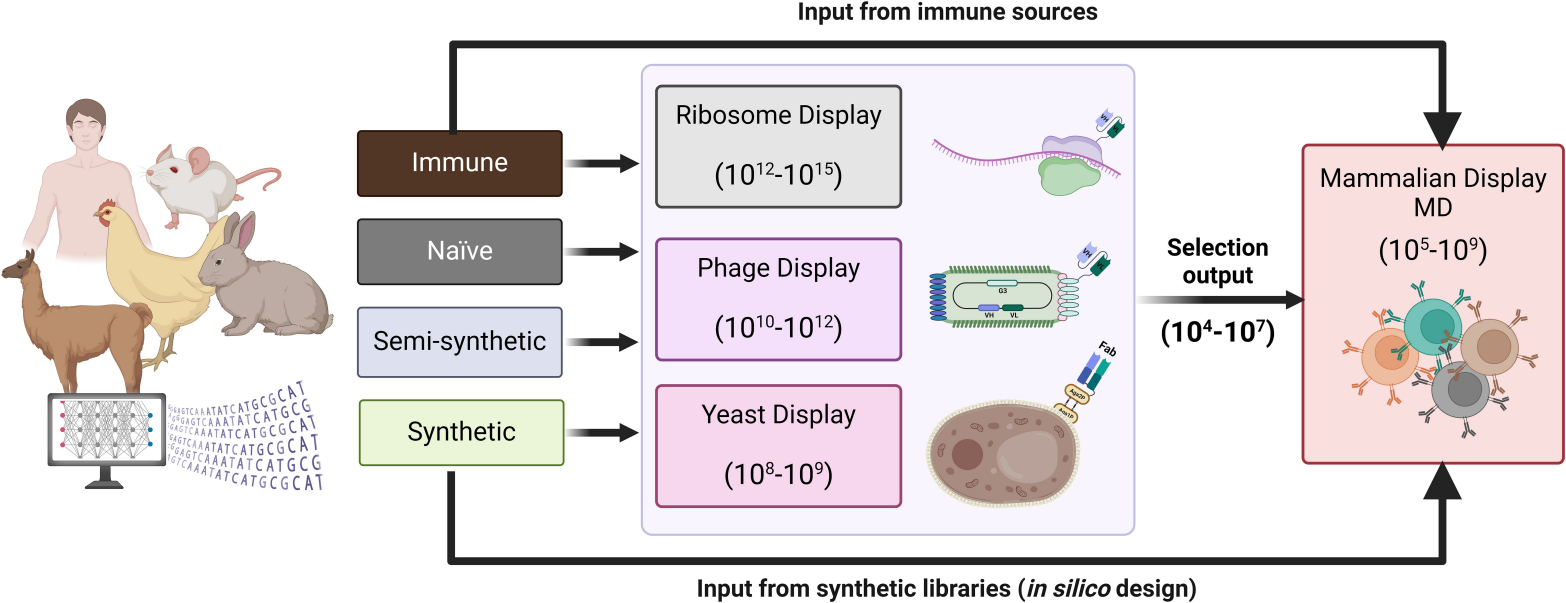
Antibody diversity introduced into mammalian display libraries. Antibody repertoires from immune, naïve, semi-synthetic or synthetic sources. Typically, naïve or semi-synthetic repertoires expressed in phage, yeast or ribosome display can undergo a few rounds of selections, and the resulting focused library can be integrated into a mammalian display platform for further screening in the final therapeutic format. The diversity of immune and synthetic libraries (designed *in silico* for antibody engineering with libraries up to 10^7^) can be covered in mammalian display systems without needing pre-selection via other *in vitro* display technologies.

**Figure 3:**
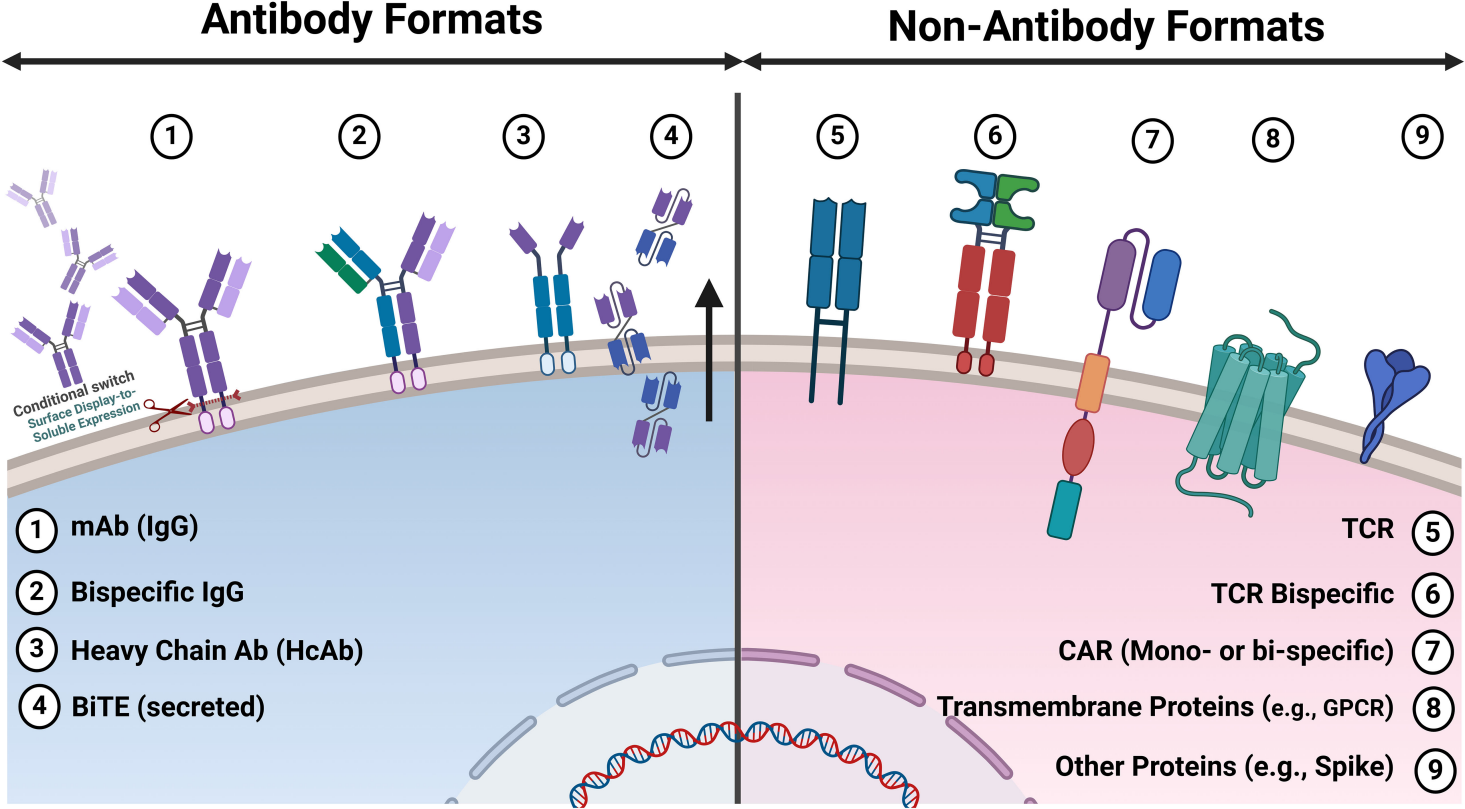
Antibody and non-antibody formats displayed on mammalian cells for discovery applications. Various antibody and non-antibody formats reported on mammalian cells for discovery applications. 1. IgG: Both surface display and conditional switching to soluble expression/secretion enable functional screening via microfluidics. 2. Bispecific IgG: Antibody repertoires engineered to bind two different antigens or epitopes, enhancing their therapeutic potential. 3. Heavy Chain Only Antibodies (HcAb): Derived from camelids or semi-synthetic libraries, these antibodies are smaller and can access epitopes that conventional antibodies cannot. 4. BiTE (Tandem scFv): Bispecific T-cell engagers that link T-cells to cancer cells, promoting targeted immune responses. 5. TCR: T-cell receptors that recognise peptide antigens presented by MHC molecules, crucial for adaptive immunity. 6. Bispecific TCR: Engineered TCRs that can simultaneously recognise two different antigens, improving specificity and efficacy. 7. CAR: Mono- or Bispecific Chimeric antigen receptors (e.g. scFv or VHH) are synthetic receptors that redirect T-cells to target specific antigen(s) on cancer cells. 8. Transmembrane Proteins: Complex multi-pass membrane proteins Such as G protein-coupled receptors (GPCRs) are essential for numerous physiological processes and drug targeting. 9. Other Protein: Includes surface protein antigens from pathogens used for vaccine development and therapeutic targeting (e.g., spike protein from SARS-CoV-2).

Mammalian display has been effectively combined with other display methods for antibody discovery and optimisation (**Figure 2**). For example, entire outputs from phage display can be converted into a mammalian display library, combining multi-parametric FACS-based selection in the final drug format via mammalian display (50, 56, 98). Situating mammalian display downstream of technologies that generate diverse repertoires of antibody binders maximises the available library size and enables deep mining of focused libraries, for example, from naïve (non-immune) sources. The diversity from immune repertoires can be more readily covered directly in the mammalian display system from immunised animals (99) and human immune sources (100). Direct use of fully synthetic library designs has also been reported (50) (**Figure 2**). This concept has been applied to a semi-synthetic and modular scFv phage library of developable antibody scaffolds that can be transferred to mammalian display for screening in final format [(https://fjbio.com/services/explorer-library, date accessed: 22nd of July 2024)]. Mammalian display technology can be applied beyond the antibody discovery field and has empirically validated capability for engineering multiple protein types and biologics modalities (**Figure 3**).

### 2.1 “Getting genes in”: genetic engineering and construction of mammalian display libraries

**Figure 4:**
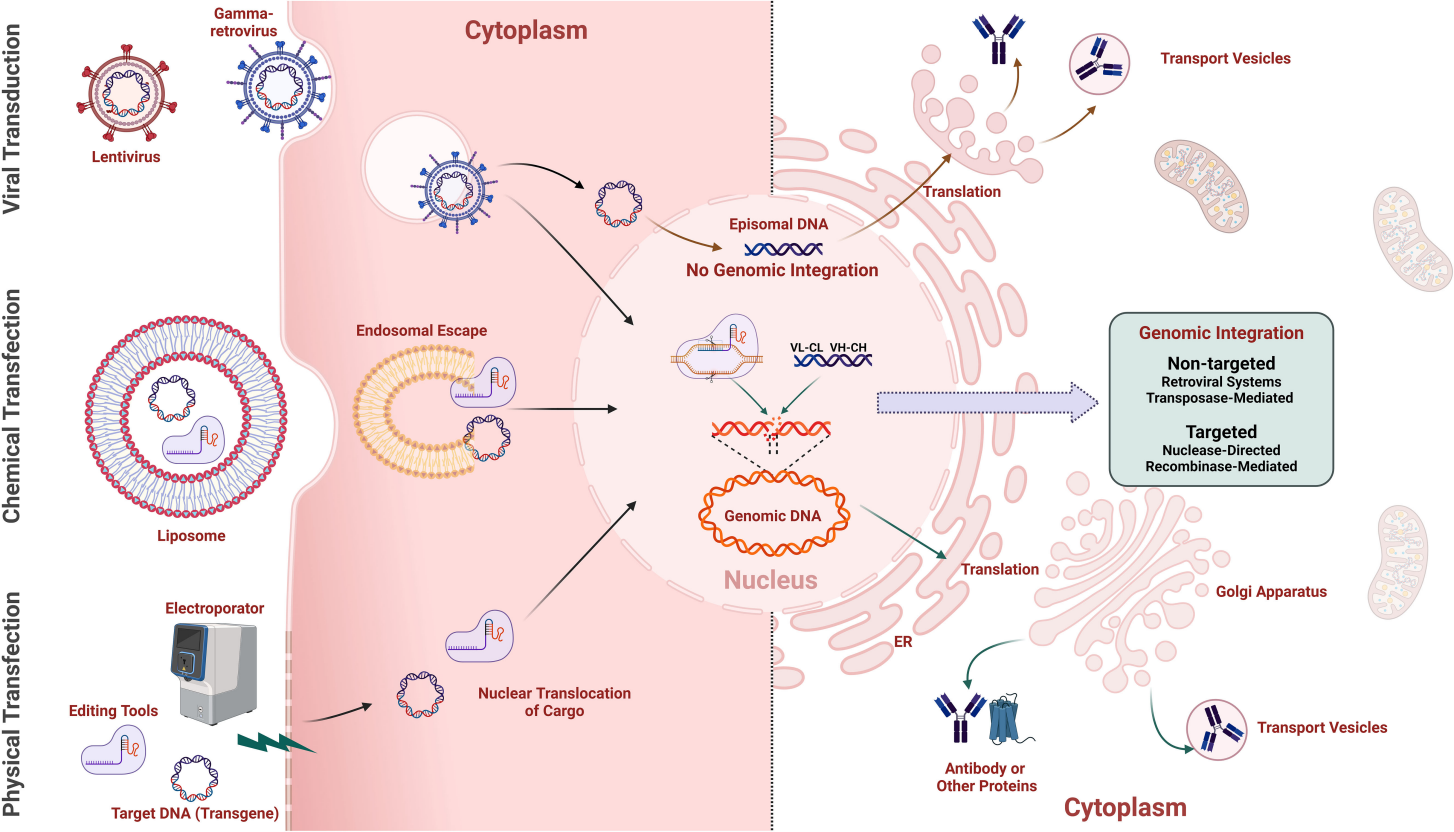
Methods for gene-editing cargo delivery and transgene integration in mammalian cells. The Left half illustrates various methods for delivering gene-editing cargo into mammalian cells for transgene delivery, including retroviral vectors (e.g., gamma retroviruses, lentivirus) using the natural infection mechanisms of viruses to deliver genetic material efficiently into host cells, chemical transfection employing lipid-based reagents (e.g., lipofectamine) to encapsulate and facilitate the entry of cargo into cells, and physical transfection, such as electroporation, using electrical pulses to create temporary pores in the cell membrane, allowing gene-editing cargo to enter the cell. The right half shows non-integration-based expression of transgenes via episomal vectors, and methods for non-targeted and targeted integration of transgenes into the genome, with the translated transgene product (e.g., antibody) being secreted from the Golgi apparatus.

The construction of stable mammalian display libraries requires efficient delivery of genetic material into mammalian cells coupled with transgene insertion into the host genome or retention by other means for stable library construction. Introducing DNA into higher eukaryotes is more challenging than in bacteria and yeast, and libraries are typically smaller for the same input DNA (**Table 1B**). Early approaches used chemical transformation (101–105) with modest results. Transient expression systems have also been reported but have clear limitations in genotype-phenotype coupling and are reviewed elsewhere (51). More recently, library construction has been transformed by efficient gene delivery using electroporation or by introducing viral vectors (**Figure 4**).

Gene delivery efficiency is directly related to the achievable library size for a given approach. Alongside efficient gene delivery is the requirement to ideally insert a single gene per cell (“monoclonality”) to enable efficient enrichment of clones with the desired properties. Trade-offs between gene insertion efficiency and genetic synteny/monoclonality affect library size and quality. Thus, the methodology employed for cell line engineering determines multiple aspects of performance and suitable applications for mammalian display systems. The available methodologies differ in efficiency, ease of use, the process modifications needed to ensure a single gene per cell, the size and complexity of genes that can be delivered, and the range of compatible mammalian cell lines. These features, in turn, dictate the mammalian display system’s functional attributes. Integration of libraries of antibody genes into the genome may be broadly grouped depending on whether integration is non-targeted or targeted (**Figure 4**). Several other approaches not dependent on integrating gene libraries are also discussed.

### 2.2 Getting genes in: non-targeted genomic integration

**Figure 5:**
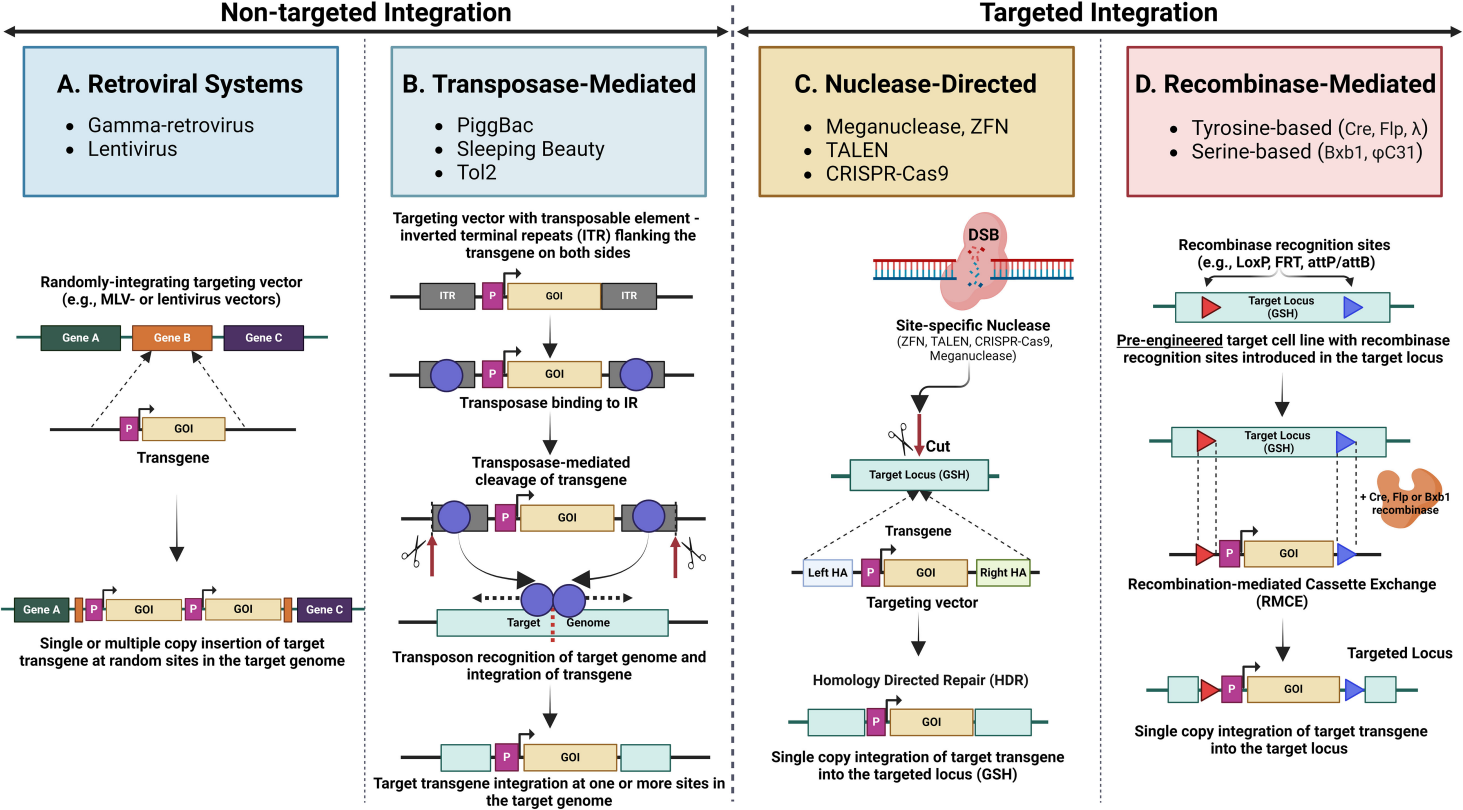
An overview of common non-targeted and targeted integration mechanisms. Non-targeted integration methods include **(A)**. Retroviral systems, which integrate transgenes into the host genome randomly via retroviruses (gamma retrovirus, lentivirus) and are often used for stable gene expression; **(B)**. Transposase-mediated non-targeted integration, where transposases (e.g., PiggBac, Sleeping Beauty, Tol2) mediate the insertion of transgenes at random locations within the genome. Targeted integration methods include. **(C)**. Nuclease-directed integration utilising engineered nucleases (e.g., ZFNs, TALENs, CRISPR/Cas9) to create double-strand breaks (DSBs) at specific genomic loci, promoting the efficient insertion of transgenes at precise locations. **(D)**. Recombinase-mediated integration employing site-specific recombinases (e.g., Cre, Flp, Bxb1) to facilitate the insertion of transgenes into pre-engineered cells containing engineered recombinase recognition sites in the target loci.

One approach to efficient random integration is using viruses that integrate their genetic information. For example, retroviruses are enveloped RNA viruses that are reverse transcribed, with the resulting DNA being integrated into the genome of dividing cells. Retrocyte display represents an early example of the use of a vector based on gamma-retrovirus, such as the murine-leukaemia virus (MLV), to deliver antibody libraries into a B cell line (106) (**Figures 4**, **5A**, and **Table 2**). This technology has been successfully implemented in mAb development, resulting in multiple clinical-stage molecules (**Table 2**). Lentiviruses are a widely used sub-group of retroviruses with the added benefit of infecting non-dividing cells (**Figures 4**, **5A**, and **Table 2**). These viral vectors offer efficient transgene delivery, and transduction methods require careful optimisation to ensure a sufficient level of monoclonality (i.e. the proportion of cells harbouring a single gene) (15, 18, 106, 120–122). Lentiviral delivery is often optimised by applying a low ratio of the virus to target cells with a multiplicity of infection (MOI) around 1 (15, 22, 108). Effective transduction via a slightly elevated MOI (of 2) has been combined with titration of coding into non-coding lentivirus to ensure an even distribution of coding lentivirus and, ultimately, high monoclonality (123). However, the pseudo-random nature of lentiviral integration can lead to variable transgene expression and insertional mutagenesis, negatively impacting the quality and consistency of the resulting libraries (124). Lentiviral vectors prefer integration into intra- or intergenic regions of the genome, leading to differences in transgene stability and complicating applications requiring uniform gene expression across the cell population (125). These non-targeted integrations can result in multiple transgene copies at different loci, causing recombination events or gene silencing due to the surrounding chromatin environment (126).

**Table 2:** Comparison of mammalian display technologies used in therapeutic antibody discovery.

	Retrocyte display	Lentiviral mammalian display or secretion systems	Transposase-mediated integration	Nuclease-directed integration	Recombinase-mediated integration	Episomal IgG expression and in-cell diversification via AID	In-cell diversification via RAG1/2 and RSSs
**Gene delivery method**	Gamma retrovirus	Lentivirus	Electroporation	Electroporation	Lipofectamine	Fugene, Lipofectamine	Lipofectamine
**Cell lines reported**	Murine pre-B cells	CHO-S, HEK293 T	Murine pre-B cells	HEK293, CHO, Jurkat	CHO	HEK293 C18, Epi-CHO	HEK293
**Requires engineered cell line**	Yes	No	Yes	No	Yes	No	Yes
**Single locus**	No	No	No	Yes	Yes	No	Yes
**Integration method**	Viral transduction	Viral transduction	Transposon	Nuclease	Bxb1 integrase	Episomal	N/A
**Integration efficiency**	<5%	0.5-5%	6-7.5%	0.5-5%	1.7%	N/A	N/A
**Associated Companies**	4-Antibody (now Agenus)	HiFiBio, Memo Therapeutics, Merck KGaA	NBE Therapeutics	Iontas/FairJourney Biologics	Orion	AnaptysBio	Innovative Targeting Solutions (HuTARG™)
**Reported library sizes**	10^6^ - 10^8^	10^6^	10^6^	10^6^- 10^8^	10^6^ – 10^8^	N/A	>10^8^
**Cargo size**	<7 kb	<7 kb	At least 10 kb	At least 10 kb	At least 10 kb	At least 10 kb	At least 10 kb
**Modular input**	Yes	Yes	Yes	Yes	Yes	No	No
**Reported number of mAbs in clinic**	5 (Botensilimab, Balstilimab, Agen 2373, Incagn2390, MK4830)	1 (antiBKV)	1 (NBE-002)	3	Not reported	3 (Rosnilimab, ANB032, ANB033)	Not reported
**Advantages**	Cryopreserved cellular antibody library available for screening.	Simultaneous display and secretion.	Favours transcriptionally active loci. No specialist equipment is needed.	Targeted integration. Transcriptional normalisation. Validated developability prediction. Cell line agnostic.	Targeted integration. Transcriptional normalisation. Validated developability prediction.	Diversification *in situ.*	Diversification *in situ* reduces tissue culture bottleneck on library size.
**Disadvantages**	Random integration leads to variation in transcriptional levels	Random integration leads to variation in transcriptional levels	Non-targeted integration leads to variation in transcriptional levels	Requires design and validation of nuclease	The two-step process requires a stable cell line with a landing pad plus RMCE	No genomic integration	Least flexible. Input requires bespoke cell lines. Limited control of library design.
**Selected references and issued patents**	(106, 107)	(15, 108–110)	(52, 111)	(50, 56, 112, 113)	(53, 114, 115)	(99, 116, 117)	(55, 118, 119)

The limited DNA cargo achievable by viral delivery methods (less than 10kb) creates a constraint in the compatible genetic elements and, thus, the complexity of binder formats that can be accommodated (127). Approaches to circumvent these problems include using small antibody fragments (122) and the sequential delivery of heavy and light chains (15, 22, 106, 108, 123). Another aspect to consider for mammalian libraries produced through lentiviral delivery is the optimal timing for selection and screening to prevent potential display loss caused by CMV promoter silencing, as noted by Sadelain et al. (128).

As an alternative to viral integration methods, antibody gene libraries can be integrated using transposons (**Figures 4**, **5B**, and **Table 2**). Transposons are mobile genetic elements that facilitate the “cut and paste” insertion of DNA sequences into the genome via a transposase enzyme. This system allows random integration of transgenes by flanking the desired gene with transposon recognition sequences and co-delivering it with transposase-encoding nucleic acid. This approach is exemplified by developing antibody libraries using the “PiggyBac” transposon system (52, 129). Other transposon systems, such as Sleeping Beauty and Tol2, have also been used for genomic integration of transgenes in mammalian cells, though not specifically for antibody library generation, with varying transposition efficiency depending on the cell type (130, 131).

However, transposon-based systems, including PiggyBac, Sleeping Beauty, and Tol2, have limitations. The quasi-random nature of transposon-mediated integration can lead to insertional mutagenesis, where transgenes disrupt essential genes or regulatory regions, potentially causing unintended effects. Additionally, like lentiviral integration, the lack of control over insertion sites can result in variable transgene expression, influenced by the surrounding genomic context. The stability of integrated transgenes may also be compromised if the transposase enzyme remains active, leading to potential remobilization and loss of the transgene (131). These factors present challenges in achieving consistent and stable transgene expression in mammalian display libraries.

### 2.3 Getting genes in: targeted genomic integration

Mechanisms for stable transgene propagation include nuclease-directed integration and recombinase-driven approaches. In contrast to random gene insertion methods, site-directed approaches target a defined locus and ensure a “transcriptional normalisation” of clones in the resulting mammalian display library. The different site-specific integration methods described below are schematically illustrated in **Figures 5C, D**.

#### 2.3.1 Nuclease-directed integration

Undirected transfection of libraries of antibody genes into mammalian cells results in random integration of multiple different antibody genes into the genome of each cell. Homologous recombination, in contrast, directs single copies of incoming DNA to a specific locus within the genome. Historically, this has been achieved by flanking the incoming DNA with long homology arms (approximately 5kB each) to direct the incoming DNA to the homologous genomic locus. This approach was used by Melidoni et al. (2013) (132) to introduce libraries of antibody genes into mouse embryonic stem cells to identify functional blockers of differentiation. While this original approach to homologous recombination benefits from single gene integration at a fixed locus, it is inefficient. Porteus et al. (2003) (133) demonstrated that cleavage of the genome by specific nucleases can significantly increase the efficiency of homologous recombination and permit significantly shorter homology arms. This approach was initially demonstrated using meganucleases and was subsequently extended to zinc finger nucleases and TALE nucleases (134). Zinc finger nucleases and TALE nucleases require more complex design and construction of sequence-specific nucleases. The capability for targeted genomic cleavage to assist homologous recombination was significantly facilitated by the introduction of RNA- guided cleavage using CRISPR/Cas9 systems (134). Parthiban et al. (50) took advantage of the efficiency of this nuclease-directed approach to generate mammalian display libraries of over 10^7^ clones (**Figure 5C**, **Table 2**). Parola et al. (135) also demonstrated this approach to library construction. In summary, nuclease-directed integration provides a convenient method for creating large antibody display libraries in mammalian cells with the added advantage of transcriptional normalisation through single-site integration. Since targeting is determined by the homology arms of the incoming donor and the nuclease specificity, there is no need to pre-engineer the target cell. This approach can, therefore, be easily applied across multiple cell types.

#### 2.3.2 Recombinase-mediated integration

Recombinases catalyse genomic insertion of a DNA sequence flanked by a pair of sequence motifs (e.g., attP/attB) into an engineered cell line that harbours corresponding recombinase recognition sites. Early developments in the use of recombinases include recombinase-mediated cassette exchange (RMCE) to allow targeted transgene insertion at a predetermined genomic locus (i.e. the “landing pad”) to generate libraries with one gene per cell and without induction of a double stand break (DSB) (136) (**Figure 5D**, **Table 2**). RMCE is often applied and further developed to enhance the productivity of single therapeutic antibody lead candidates in production cell line engineering (137, 138), but similar principles have also been applied to create mammalian display libraries (139).

Site-specific recombinase systems consist of two groups: serine recombinases (e.g., ϕC31 and Bxb1) and tyrosine recombinases (e.g., Flp, λ and Cre) (140). These groups have different recombination mechanisms, but both rely on recognition sites in the host to enable DNA excision and repair (141). Flp recombinase has been the subject of extensive research and application, exemplified by Zhou et al. (142). Initial low integration efficiency was enhanced among other approaches by designing a mixed Cre/Flp integration setup for “dual RMCE” (104). Bxb1 recombinase previously had been effectively employed in production cell line engineering (143, 144) and identified as very specific and efficient early on by Xu et al. (145). Upgraded Bxb1 recombinase-based systems were recently used successfully for mammalian library construction, with several teams (53, 114, 121) applying the system developed by Chi et al. (146). While Flp-based methodologies yield 1% or lower or lower integration efficiencies and library sizes up to 1 million, Bxb1 utilisation allowed 1.7 - 38% site-specific integration (53, 114) and reduced efforts for larger library sizes that were instead limited by screening throughput capacities (114) or DNA input diversity (53). Matreyek et al. (147) describe an improved lentiviral landing pad (LLP) system that simplifies the generation of new landing pad cell lines and enhances recombination efficiency by incorporating the Bxb1 recombinase within the landing pad itself. They also introduced positive and negative drug selection markers, facilitating the enrichment of recombinant cells and enabling the use of larger libraries. A recent optimisation of enzyme-encoding plasmids yielded enhanced nuclear localization and increased stable integration efficiency that may further facilitate larger library generation and underline the current surge of advancements for Bxb1-based RMCE (115). Introducing negative selection markers to eliminate cells with unwanted recombination events after cassette exchange ensures homogeneity of stable library pools (53, 114, 146). Finally, Durrant et al. (148) reported advancements beyond Bxb1 by systematically discovering recombinases for efficient large integration cassette exchange.

Further evolution reported for production cell line engineering may inform and enhance mammalian library developments. For instance, Xu et al. (136) introduce a dual site-specific integration (SSI) system in CHO cells using Bxb1 recombinase to enhance the stability and efficiency of secreted biotherapeutic expression. The system includes two independent Flp or Bxb1 loci, each equipped with a unique landing pad, improved fed-batch performance attributes and maintained stable expression profiles over extended generations. Cautereels et al. (149) developed a set of orthogonal LoxPsym sites with high specificity and minimal cross-reactivity that enhance the capabilities of the Cre-LoxP recombination system for multiplexed genome engineering. Finally, Roelle et al. (150) identified orthogonal Bxb1 recognition sites to create double landing pad cells they utilised for functional characterisation in protein variant screens. The typical genomic integration efficiency rates reported using recombinase-directed approaches are below 5%. To improve the efficiency of integration, Chen et al. (151) developed an arrest RMCE (aRMCE) method with optimised conditions for cell cycle arrest and synchronisation for better temporal coordination needed in DNA recombination using dual RMCE. They report 20% integration efficiency of the target gene and display monoclonal libraries containing over 10^7^ NGS-verified Fc variants as IgG to identify variants showing enhanced binding to specific Fc gamma receptors (FcγRs) and improved effector cell functions. This RMCE technique developed for arresting the cell cycle can be applied to various cell clones, target transgenes, transfection methods, and cell types. Recent advancements underline a continuing evolution of recombinase technologies and their expanding role in mammalian library applications.

### 2.4 Other approaches to library construction

Alternatives to creating stable mammalian display libraries by genomic integration are outlined below.

#### 2.4.1 Episomal propagation

Modified mammalian cell lines carrying viral elements and complementary plasmid modifications can be combined to achieve extrachromosomal plasmid amplification and persistence in transfected cells (152–154). This technology was initially developed to enhance recombinant protein expression systems, but several groups have employed derivative methods to create mammalian display libraries (116, 117). Dilution of antibody encoding plasmid DNA (117) and/or low copy number episomal systems (54) have been used to discover and optimise mAbs. The copy number of the episomal-maintained plasmids varies substantially depending on the molecular elements used (53, 144, 145). Methods with the lowest achievable levels are preferable for mammalian display applications. For example, Bowers et al. report 3-5 plasmid copies per cell for each of the heavy and light chain plasmids, which are carried on separate vectors (114). The limitations of episomal approaches to mammalian display are like those described above for non-targeted integration methods, specifically the innate “trade off” between “monoclonality” and transformation efficiency.

#### 2.4.2 “In-cell” diversification

An alternative to cloning and incorporating exogenous repertoires is the direct *in situ* generation of novel genetic variants (55, 99, 116, 155). Here, a “base” cell line is engineered such that clonal diversity is created by novel genetic rearrangements/mutagenesis in each cell upon induction of bespoke genetic machinery.

**Figure 6:**
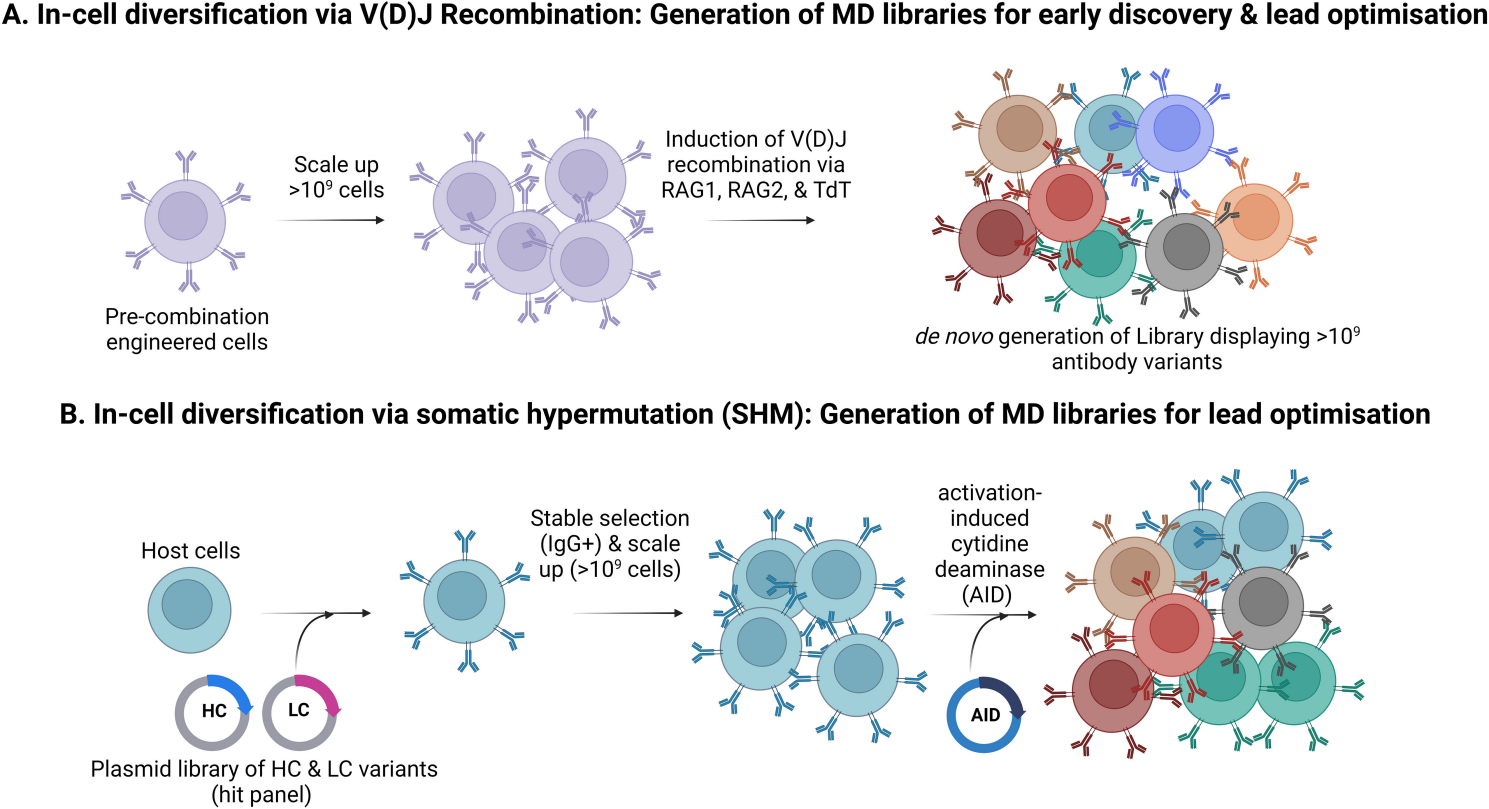
In-cell diversification to create mammalian display libraries. **(A)**. Generation of antibody repertoires by *in situ* V(D)J recombination. This process generates antibody diversity that mimics the immune system and circumvents the need for efficient transgene insertion but is incompatible with modular library input. **(B)**. Generation of antibody repertoires by Somatic Hypermutation (SHM) This strategy employs SHM to create antibody diversity. Here, heavy and light chains from selected target-specific hits are introduced into host cells (e.g., HEK293), which are then selected for stable IgG expression and scaled up. Activation-induced cytidine deaminase (AID) is then induced to trigger SHM.

Generation of initial antibody diversity in B cells involves combinatorial recombination of germline-encoded V, D and J segments to create diversity (**Figure 6A**). This is extended further by somatic mutation coupled with affinity selection within germinal centres (156). Gallo et al. (118) describe a mammalian display system that uses this same molecular machinery mediating the recombination of V(D)J gene segments within an engineered HEK293 cell line. The cell line harbours antibody gene recombination signal sequences (RSSs) and inducible RAG1 (recombination-activating gene 1) and RAG2 (recombination-activating gene 2) to enable a unique V(D)J rearrangement to occur *in situ* in each cell upon induction. This can be performed in the presence of terminal deoxynucleotidyl transferase (TdT), which, together with double-strand break repair, facilitates imperfect joining of the V(D)J segments and further diversification of antibody sequences (118). This approach can also be applied to affinity maturation, although each campaign requires the generation of a bespoke cell line containing the parental antibody with RSS insertions in the CDRs (55).

Direct generation of antibody diversity within a mammalian display platform can also be achieved by mimicking the natural process of somatic hypermutation (SHM) (**Figure 6B**). Bowers et al. describe activation-induced cytidine deaminase (AID) elements for CDR-directed mutagenesis (54, 99, 116). Further enhancements in the efficiency of AID-based systems have been described using engineered derivatives of the AID enzyme and optimised target gene sequences (157). The AID system has been successfully commercialised and multiple antibodies are currently in clinical trials using this technology (**Table 2**).

Both in-cell diversification approaches mentioned above can reduce the volume of cell culture needed to maintain mammalian display libraries (approximately 10-fold) by uncoupling the efficiency of gene delivery from the introduction of clonal diversity mimicking to some extent, the *clonal diversification* in B cells as a part of the adaptive immune response. However, they are innately less flexible and modular than approaches that directly introduce gene libraries into mammalian cells. Systems that rely on direct transformation of mammalian cells to create display libraries (for example nuclease-directed or recombinase-mediated integration or viral transduction) can typically accommodate input diversity from any source (e.g., cloned immune repertoires, fully synthetic library designs, or output populations from phage or ribosome display). However, the library size is constrained by the efficiency of single-copy gene delivery and the scale of tissue culture facilities available. By contrast, *in situ* approaches allow minimal control over the input library design but reduce the tissue culture volume required to accommodate a given library size.

### 2.5 “Getting biologics out”: applications of mammalian display technology

Having constructed a library, discovery and optimisation workflows involve enrichment of binders, *in vitro* functional testing, and assessment of biophysical attributes to identify molecules suitable for pre-clinical development. Considerations for determining the optimal screening cascade include the availability, predictive power, and throughput of screening assays, and the anticipated frequency of the desired properties in binder populations. The following sections describe the application of mammalian display to various drug discovery workflows.

#### 2.5.1 Mammalian display for selection of binders

Cell surface display of biomolecule libraries can be achieved by direct anchoring of variants via insertion of a transmembrane domain (most commonly PGDFR) or indirect anchoring through the expression of a “capture” partner protein (18, 158–162), which will be discussed later in detail. Enrichment of cell-displayed antibodies typically uses FACS to probe antigen binding. This allows the selection of molecules based on their binding properties (e.g., affinity to target, recognition of species orthologues and specificity against human homologues). The benefit of multi-parametric flow sorting with mammalian display is broadly comparable to yeast display (44, 163) and is also widely reported elsewhere (50). Once binder populations with the desired characteristics have been enriched, recovery of clones of interest can be achieved in several ways. For example, individual cells can be sorted and grown clonally, and genetic information can be retrieved by PCR (or single-cell RT-PCR immediately after FACS). Alternatively, the enriched population of interest can be used directly for sub-cloning and production and/or sequencing to identify monoclonal “hits”.

Alternative approaches have been described wherein antibodies are secreted and “self-labelled” by binding to target antigens expressed on the same cell (122). This approach can, however, be limited by crosstalk between antibody-secreting cells. Zhang et al. (164) describe a functional screen using a combined production/reporter cell wherein an extended linker tethers the antibody to enable autocrine activation of a target receptor on the same cell. Whilst antibody display combined with FACS provides a powerful tool for interrogating binding properties and can be adapted for some functional applications, many screening methods of function and potency require soluble antibodies. Thus, switching between membrane-tethered and secreted formats is advantageous for integrating mammalian display with high-throughput functional screening technologies. The application of mammalian display to facilitate early functional screening and associated enabling technologies are discussed below.

#### 2.5.2 Mammalian display for selection on developability and biophysical properties

Historically antibody drug development has been focused on the affinity, specificity and functionality (e.g., cytokine neutralisation) of antibody lead compounds. More recently, there has been a realisation of the additional need to identify antibodies with optimal biophysical properties (85–88, 165). This has been driven by clinical failures and the increasing use of subcutaneous administration, which requires antibodies to be formulated at high concentrations, e.g., >100 mg/ml. The measurement and assessment of biophysical properties was historically left until late in the development process but is increasingly being incorporated earlier during candidate selection.

Measuring melting temperature and comparing expression yield have been used as surrogate indicators of developability. Antibodies with a Tm approaching physiological temperature are more liable to unfold with consequent aggregation and are unlikely to be good development candidates (86). This is, however, a low bar and having an acceptable Tm is no guarantee of optimal developability properties, especially those relating to colloidal stability (i.e., the behaviour of folded protein in solution). A good development candidate would be expected to give a high production yield (e.g., expressing >1g/litre equivalent to 1mg/ml), but passing this “filter” again does not guarantee success in the development pipeline. For example, Dobson et al. (166) describe MEDI1912, which has a favourable Tm and good expression in transient culture, which nonetheless suffers from poor biophysical properties at higher concentrations, leading to aggregation and polyreactivity.

Thus, while high-yielding antibodies may achieve 1mg/ml in tissue culture supernatants, undesirable properties such as aggregation and high viscosity can be driven by the self-association of molecules (irrespective of thermostability), and this may only become apparent when those antibodies are formulated at higher concentrations. Similarly, the propensity to bind other molecules with low specificity (polyreactivity) may become more apparent at higher concentrations. This, in turn, can require producing relatively large amounts of antibodies to detect sub-optimal biophysical properties effectively. Several assays have been developed to measure these undesirable molecular cis or trans interactions, i.e. aggregation or polyreactivity (86, 87, 167).

In a seminal publication, Dyson et al. (56) described the ability of mammalian display to detect differences in biophysical characteristics, enabling the selection of biophysically improved variants from mammalian display libraries. Using pairwise comparisons of antibodies with similar high titre transient expression profiles but differentiated biophysical properties, they showed strikingly different display levels (of up to a 2-log difference in fluorescence intensity) between molecules with “good” and “poor” self-association propensity.

Moreover, this separation of molecules based on determinants of colloidal stability, such as self-association propensity, is largely absent when the same protein pairs are displayed on yeast. Yeast display relies on linkage to the cell wall rather than the plasma membrane, so this difference in sensitivity between mammalian and yeast display systems suggests a mechanism reliant on the dynamics of protein presentation on the mammalian plasma membrane (discussed below). The ability to discriminate between molecules having different biophysical properties based on display level has enabled direct enrichment for superior developability characteristics. The progress of antibodies discovered using the nuclease-directed and RMCE mammalian display approaches implies a correlation between display level and behaviour in Chemistry, Manufacturing and Controls (CMC) processes and accelerated transition through CMC and into the clinic (**Table 2**).

**Figure 7:**
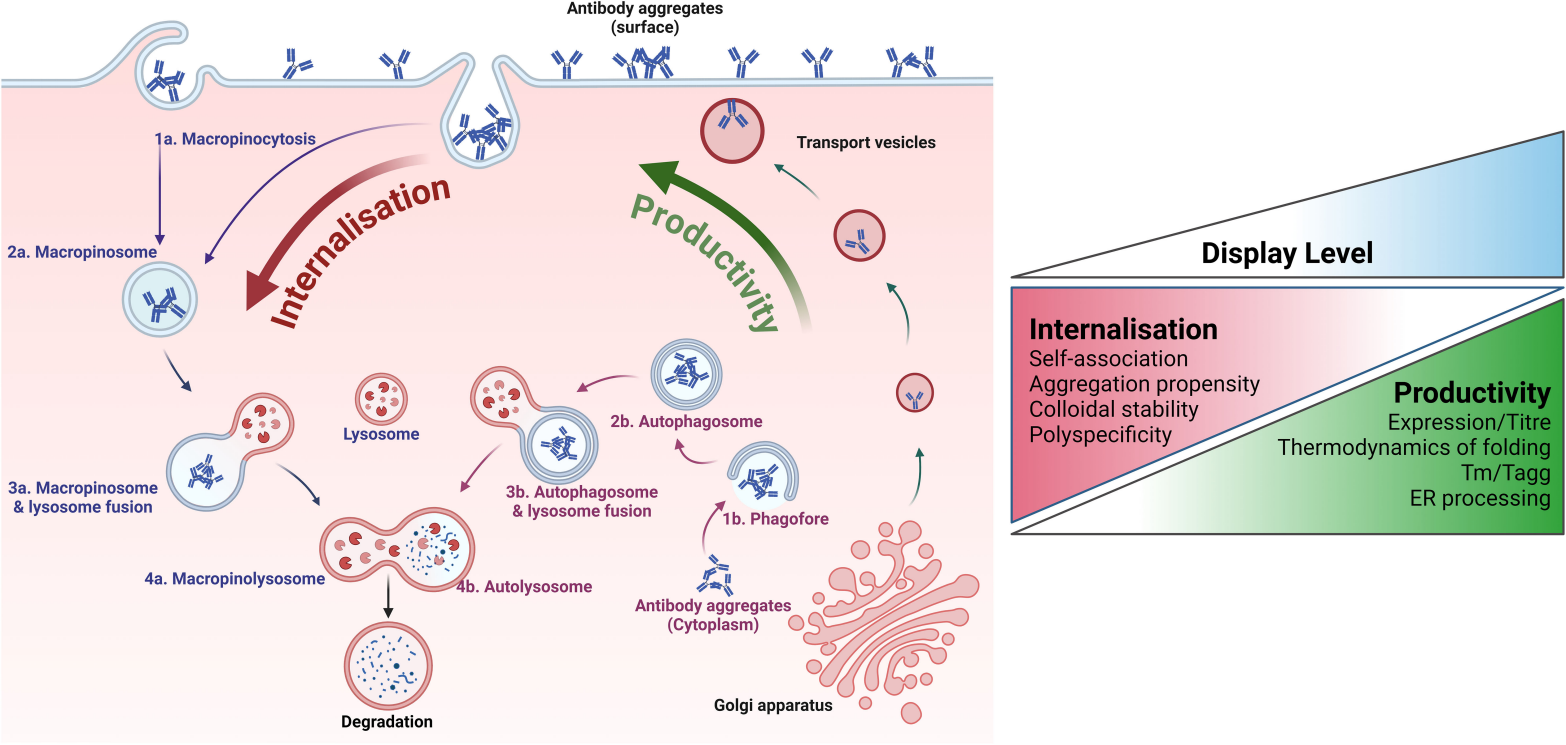
Relationship between mammalian cell surface display and developability properties. Key quality control (QC) mechanisms employed by mammalian cells to correct the expression of aggregated or sub-optimal antibodies are illustrated. The overall developability of an antibody emerges from various distinct biophysical characteristics with different impacts on the molecule’s manufacturability, stability, and biological tolerability. These features relate to different aspects of the mammalian protein homeostasis machinery. Assuming stable site-specific integration of antibody genes at a single locus, biophysical properties that correlate with productivity (and secreted antibody titre) include aspects of thermostability and folding and early onset aggregation around the ER or during trafficking to the cell surface. The accumulating antibody must then be maintained at a high local concentration on the plasma membrane. Under these conditions, molecules exhibiting sub-optimal biophysical properties are internalised and degraded faster than more stable ones. The importance of the internalisation rate in determining the overall display level of an antibody in the mammalian system is underscored by multiple publications reporting differential presentation levels for clinical-stage antibodies that share high productivity rates but differ in other biophysical attributes. Crucially, the internalisation rate appears to be correlated with critical biophysical properties in solution, such as self-association propensity and polyreactivity.

Dyson et al. (56) propose a mechanism whereby the display level of each molecule is determined by two parameters: (i) the rate of synthesis and trafficking to the plasma membrane and (ii) the rate of internalisation and removal from the cell surface. The relative rates of these components determine the equilibrium position and display level (**Figure 7**). In contrast to expression systems based on antibody secretion, mammalian display retains produced antibodies in the low volume perimeter encircling the plasma membrane and, therefore, achieves very high local concentration. This likely forces library members through a concentration “bottleneck” early in the discovery process with the self-association of non-optimal clones at high concentration driving a higher internalisation rate. Hence, the internalisation rate is a sensitive reporter of key biophysical attributes required for drug-like colloidal stability properties. This feature is unique to mammalian display systems and is distinct from expression-based measures, which typically correlate with other molecular characteristics, such as the thermodynamics of folding/unfolding. The mechanism proposed by Dyson et al. (56) has gained support from more recent work showing an increased rate of micropinocytosis when surface aggregation of antibodies is induced (168).

Dyson et al. used nuclease-directed integration to target a single locus. While this association of biophysical properties to display level in mammalian cells could apply to the different library-building approaches described above, targeted integration has the added benefit of achieving transcriptional normalisation, meaning that differences in antibody presentation level are primarily related to the properties of the protein itself. The work of Huhtinen et al. (53) extends the above observation using targeted integration of library members by Bxb1 recombinase followed by selection based on presentation level. Gaa et al. (109) also demonstrate the separation of clinical antibodies exhibiting “good” and “poor” developability after site-specific integration.

The mechanisms of protein quality homeostasis in mammalian cells are complex and pleiotropic, incorporating multiple pathways that operate not only during protein folding and trafficking but also on mature proteins across multiple cellular locations (168–170). The equilibrium position for display level is likely reached during cell culture, where the plasma membrane is dynamic, and staining at low temperature “freezes” the proportion of molecule at the surface before detection. Thus, experimental details such as culture and staining conditions might impact the resolution and dynamic range of selection and enrichment based on developability – i.e. the capability of the developability selection pressure will likely depend on the construction of the mammalian display platform and the process employed. Currently, there is no published “side-by-side” comparison between systems to elucidate the impact of mammalian display methodologies on the types of biophysical properties that can be interrogated and the resolution achievable. This, and the mechanistic details underpinning developability discrimination, remain an area of interest.

#### 2.5.3 Application of mammalian display to the development of non-antibody therapeutics

Mammalian display technology extends well beyond traditional antibody applications (**Figure 3**), such as discovering and optimising T cell receptors (TCRs) for therapeutic applications, providing powerful tools to target a broad array of antigens with high specificity and affinity. Early implementations of this technology demonstrated the potential to engineer TCRs that recognise peptide-major histocompatibility complex (pMHC) molecules, overcoming the limitations of natural TCR affinity and specificity (171, 172). Experimental engineering efforts have also targeted the optimisation of TCR affinity through direct mutation and selection, using technologies such as alanine scanning to identify key residues in TCR-pMHC interactions. This approach has significantly streamlined the process of generating libraries of high-avidity TCRs (173). Subsequent advancements included the development of high-throughput screening methods using microfluidics to identify potent TCRs from extensive repertoires of human T cells, thus enhancing the efficacy of TCR-based therapies for both viral infections and cancer (36).

Moreover, integrating mammalian display techniques with chimeric antigen receptor (CAR), T-cell development has enabled the fine-tuning of CAR-T cell responses to target tumours more effectively (174). Similarly, bispecific TCRs have benefited from CHO display systems, which facilitate the maturation of TCR affinity in the context of their final therapeutic format, showing increased efficacy in targeting cancer cells (139). Innovations like the TCR-Engine platform have furthered this field by engineering TCRs with enhanced potency and specificity using high-throughput genetic and computational tools, ensuring safety and efficacy in immunotherapy applications (175). Additionally, novel platforms such as those employing Signalling and Antigen-presenting Bifunctional Receptors (SABRs) have emerged for efficient TCR antigen discovery, offering scalable solutions for personalised immunotherapy (176). Bispecific CARs based on single-domain antibodies have been developed in the clinic using single-domain modules (177). Collectively, these advancements underscore the transformative impact of mammalian display on the landscape of TCR and T-cell antigen discovery, propelling forward the capabilities of adoptive cell therapies.

Mammalian display technology has also made significant strides across various translational biomedical applications, particularly in directed evolution, protein engineering, and therapeutic discovery. Mammalian display has also been used to generate peptide libraries for drug discovery and screening (178). Crook et al. describe a mammalian display platform designed to screen cystine-dense peptides (CDPs) that are challenging to produce due to their complex disulfide connectivity. The platform’s efficacy was demonstrated by identifying and engineering a CDP that inhibits the intranuclear interaction of YAP: TEAD transcriptional activators involved in the Hippo pathway, which is commonly dysregulated in many human cancers. This mammalian display system allowed for high-quality, diverse scaffold library screenings, ensuring proper folding and stability of the peptides. The study highlights the platform’s potential for rapid discovery and affinity maturation of therapeutic peptide candidates. On the other hand, platforms like Spike Display are advancing the rapid characterisation and optimisation of viral proteins, such as the SARS-CoV-2 spike protein, which is crucial for vaccine development and therapeutic antibody screening (179). Novel platforms have been developed to evolve complex receptor systems such as GPCRs within their native signalling environments in mammalian cells, essential for understanding receptor pharmacology and developing therapeutic agents (180).

Emerging mammalian cell-based directed evolution methods also address significant gene and cell therapy challenges, such as enhancing protein expression by optimising 5′ UTRs and designing more efficient delivery vectors (181, 182).

#### 2.5.4 Advancements in “function-first” screening technologies and application of microfluidics

Broad screening for function during combinatorial antibody library selection or initial hit identification early in the discovery process can shorten timelines but necessitates novel, robust, and efficient screening methodologies. Overcoming these challenges is crucial for advancing mammalian antibody display technology and realising its full potential in therapeutic and diagnostic applications. Given the substantial development of mammalian libraries in secretion mode and microfluidic-assisted hit discovery, their combination for function first antibody discovery is discussed below.

Upstream activities, such as diversity sourcing and library generation, resemble the workflows of display approaches and use both *in vitro* and *in vivo* antibody libraries (**Figure 2**). While library cloning setups differ only in the lack of a membrane anchor, library sizes are restricted (compared to MACS and FACS) due to lower microfluidic device throughput, ranging from 50,000 to a few million, depending on the device and screening setup. Such mammalian library sizes can be realised with comparatively little effort and usually capture most of the diversity from immune sources (183, 184), output from phage display selections or combinatorial libraries of known paratope combinations (15). Likewise, binder populations enriched by mammalian display based on their binding and developability properties offer an attractive input for high throughput functional screening.

Microfluidic approaches have become a routine methodology for antibody hit discovery, interrogating plasma B cells after immunisation of mostly transgenic rodents (12, 23, 185). Antibodies secreted from ASCs (plasma cells or plasmablasts) are interrogated after compartmentalisation in microdroplets (17, 186, 187), microchambers such as nanopens (19), or microcapillaries (188–190). Screening of recombinant mammalian secretion libraries is primarily based on fluorescence. Libraries have been screened for distinct specificities to complex cell surface antigens (22, 191, 192), overcoming one of the major current limitations of cell-based display technologies. The robustness of mammalian secreting cells allows for high antibody gene recovery and longer assay times, representing a considerable advantage over B cell screens. Secretion in the relevant therapeutic format and compatibility with a mammalian target or reporter cell lines offers distinct benefits over screening antibodies produced by other hosts such as yeast. While current screening methodologies support sorting by binding followed by screening for function only, the properties of mammalian libraries discussed above enable the expanded application of library-scale function-first screens, reducing the need for selection based on affinity followed by reproducing hits for functional evaluation (52).

**Figure 8:**
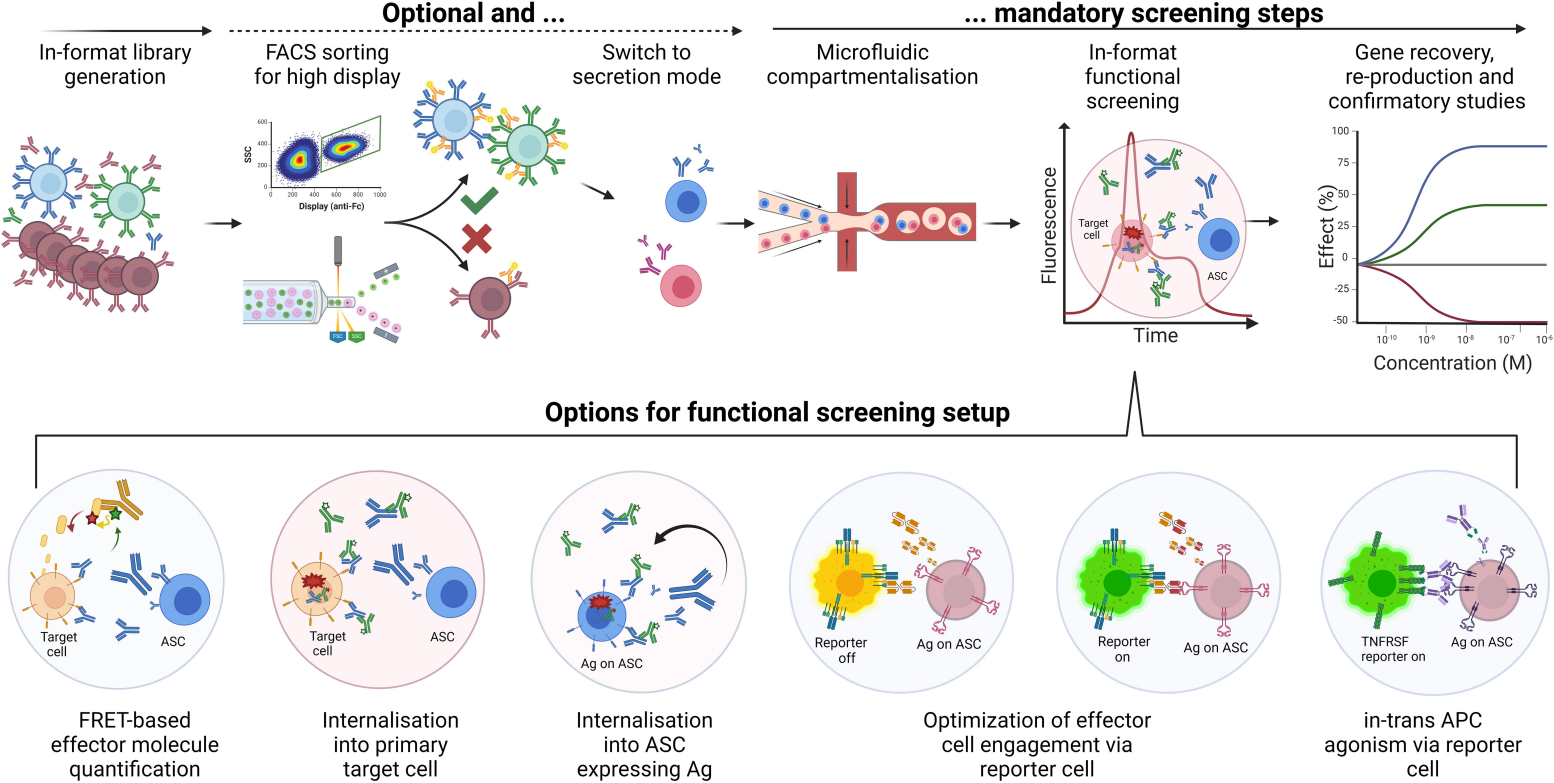
“Function-first” screening of mammalian secretion libraries. Workflow options for mammalian secretion libraries in microfluidic function-first screens, including display-to-secretion switching. Upper half. The generation of libraries in the final format can include a setup for switching from surface display to secretion. In display mode, FACS for high display level will enrich variants with suitable developability (blue and green cells). This optional step enhances library quality and increases the success rates of subsequent screening steps. Enriched or non-enriched diversities are then encapsulated in microdroplets or other microfluidic single-cell compartment modules (e.g., nanopens; not shown) and mixed with appropriate assay reagents. For example, target-positive tumour cells (orange) and pH-dependent detection (green) can be included for sorting based on internalisation rate via fluorescence peak signal. Subsequent export, gene recovery by single-cell RT-PCR, reproduction and functional evaluation can confirm functional hits (here blue versus red cell/mAb/curve). The robustness of applied mammalian cells allows for high recovery and potentially high functional hit rates. Lower half. Options for functional screening setups include (but are not limited to) quantitative detection of secondary effector molecules such as cytokines; internalisation into primary target cells or the same secreting cell for higher throughput; optimisation of T cell engager or similar effector cell recruiting bispecific antibodies using GFP-engineered Jurkat cells; in-trans target-dependent activation of TNFRSF members applying suitable GFP-engineered reporter cells.

Secretion of candidate clones in the final molecular format of the drug allows complex functional assays comprising several components and cells. In a simple “2-cells” setup, ASCs can be mixed with antigen-expressing cells for hit discovery towards target-specific internalisation utilising pH-dependent detection reagents (**Figure 8**) (123). Inspired by preceding autocrine designs of the Lerner group (120), a “1-cell” setup combining recombinant antigen expression on the same cell that secretes the antibody candidate (**Figure 8** lower half) yielded reduced background and high enrichment rates in proof-of-concept studies for internalisation screens (123). Combining antigen and antibody expression in one cell with the addition of a functional reporter cell, “3-components in 2-cells” setups enabled screening for complex modes of action such as T cell activation in therapeutic format combinatorial BiTE libraries (**Figure 8** lower half centre) (15, 114). Identification of library variants with enhanced potency and efficacy illustrated the advantage of this approach over lower throughput rational design strategies. Yet another recent proof-of-concept study used combinatorial IgG-VHH bispecific libraries in a 3-component 2-cell setup to identify *in-trans* targeted agonists to a tumour necrosis factor receptor superfamily member (**Figure 8** lower half right). Approximately 250,000 cells were sorted and agonist bispecifics could be enriched in one round from 1% to 18% (123). Further options for functional screening setups include (but are not limited to) quantitative detection of secondary effector molecules such as cytokines and *in-trans* target-dependent activation beyond TCR or TNFRSF targets.

Most microdroplet-based microfluidic devices depend on Poisson-distributed co-encapsulation of secreting and target cells, lowering the throughput, creating heterogeneity in cell ratios, and increasing assay variance. Novel methodologies such as pre-sorting for single cell-containing droplets could increase assay stability and maximise screening success rates. Additionally, GFP-based reporter cell lines can generate significant background under suboptimal assay conditions, warranting thorough reporter cell generation and assay development. Implementing assay opportunities to readouts beyond fluorescence could significantly broaden the application space and enable even more sophisticated function-first screens, such as phenotypic screens and primary cell assays. Considering advancements for sorting downstream activities, single stably integrated secretion clones, in contrast to plasma B cells, can be expanded for post-sort secondary screening of supernatants (52, 54, 193), circumventing gene recovery and reproduction, thereby shortening downstream workflows.

#### 2.5.5 Display-to-secretion switching for combined developability and functional screening

Mammalian display levels can indicate developability (18, 53, 56) while filtering diversity based on desired binding profiles, and secretion libraries support microfluidic function-first screens. Combining these advantages, an integrated display-to-secretion switchable system could add further value to hit discovery. Large display libraries could be enriched and focused via FACS to match microfluidic throughputs for whole antibodies (via anti-LC MACS) or those showing good manufacturability properties (56). Dual display and secretion were realised for “SPLICELECT” (194) and “ABELMab” (54) platforms via alternative splicing setups for transient CHO or HEK293 transfection, respectively, enabling correlated levels of displayed and secreted antibodies, thus aiding antibody engineering and cell line development. Further approaches utilised Furin enzyme (191) or ribosomal skipping via attenuated F2A peptides for display and secretion by in- and excluding a transmembrane anchor in the nascent polypeptide chain (109, 195). While switchable systems have been developed for yeast display (158–161), the “antibody-membrane switch” (AMS) via specific DNA recombination driving alternative splicing was the first mammalian technology supporting initial FACS for maximal surface antibody levels in high-production cell clones with subsequent switch to secretion for large scale production (196). Enhancing established hybridoma-based workflows, “on-cell mAb screening” (OCMS) applied an anti-rabbit IgG membrane “anchor” with the addition of recombinant rabbit-anti-human IgG as “linker” in FACS for specificity or high production (162). Adapted to recombinant mammalian libraries, a reversible autocrine antibody display was realised via induction of a pre-integrated membrane-anchored Protein A dual Z-domain fusion protein called ZZ-PDGFR-TMD (123). FACS to microfluidic functional screening proof-of-concept studies revealed distinct enrichment of a developable and target-specific internalising antibody clone, principally confirming the applicability of such workflows for antibody hit discovery (123).

## 3 Emerging transformative technologies poised to impact mammalian display

Recent technological advancements are set to profoundly enhance mammalian display platforms, particularly with precision genome engineering and the integration of artificial intelligence (AI) and machine learning (ML) in therapeutic drug discovery. Precision genome engineering now enables high-efficiency integration of large transgenes into mammalian cells, overcoming significant hurdles such as nuclear delivery and monoclonal expression. Concurrently, progress in AI and ML is transforming antibody discovery and optimisation by providing sophisticated tools for predictive modelling and enhanced design of antibody libraries. These emerging approaches promise to drive the next wave of innovation in mammalian display technology, enabling rapid, efficient, and highly targeted development of biotherapeutics.

### 3.1 Advancements in precision genome engineering for mammalian display

#### 3.1.1 Enhancing the efficiency of large sequence insertion

Recent years have witnessed a paradigm shift in the scope and application of genome engineering in mammalian cells. Developing techniques that allow high-fidelity and stable integration of large transgenes has empowered mammalian display technology for antibody engineering. However, several challenges still hinder the widespread application of modern gene editing approaches for antibody library expression in mammalian cells, which include rapid and efficient nuclear delivery of genetic cargo, transgene stability, monoclonality, and compatibility with the large DNA fragments (>1 kb) needed for antibody or antibody fragment expression. Despite recent innovations in improving the efficiency and fidelity of single-copy transgene integration, it remains a key determinant and limiting factor for the size and quality (i.e. the frequency of clones with desired properties) of mammalian display libraries.

Scalability in genome editing is critical for applications that require large pools of mammalian cells. However, the logistics of generating, handling, culturing, and screening mammalian display libraries in pools >10^9^ cells is time- and labour-intensive for labs lacking necessary automation infrastructure and high-throughput technologies. If the transgene integration efficiency is <5%, the practical size of the library that can be achieved is <10^8^. Increasing the transgene integration efficiency and utilising alternative mammalian host cells suited for high-fidelity and stable genomic integration of large transgenes can facilitate the creation of libraries exceeding 10^8^. Combined with *in vitro* (e.g., natively paired V-gene libraries generated from the B-cell repertoire via droplet microfluidics) and in silico approaches (e.g., AI/ML discussed in the next section), the quality of the library can be further improved. The field of mammalian genome engineering continues to progress at a remarkable rate, and emerging transgene knock-in and gene editing strategies with the potential to impact the size of mammalian display libraries are discussed below.

Several strategies can enhance the efficiency of homology-directed repair (HDR) repair and transgene integration via CRISPR-Cas9-enabled precision genome engineering techniques, as reviewed by Fichter et al. (197). Early studies mainly included high throughput screening of small molecules and identifying pharmacologic agents that up- or downregulate DNA repair pathways. Most recently Wimberger et al. (198) focus on enhancing the efficiency and precision of CRISPR/Cas9 genome editing, using a method called 2iHDR. They simultaneously inhibit DNA-dependent protein kinase (DNA-PK) and DNA polymerase theta (Polϴ), using a compound AZD7648 identified by a large-scale compound library screen, which improves integration efficiency by 3.5-fold to 8.6-fold in primary CD3+ T-cells with minimal indels compared to ~5% without AZD7648. Their approach significantly boosts template insertions and minimises unintended mutations by directing the DNA repair pathway choice towards HDR rather than non-homologous end joining (NHEJ). Recent strategies have attempted to control the cell cycle by using small molecules to keep cells in the S/G2 stage (199, 200) when HDR is most active (201) or restrict Cas9 expression to only S/G2/M stages of the cell cycle (202). The latest methods have also focused on cargo fusion formats that bring donor templates and DNA repair enzymes (e.g., nickases) close to the Cas9 complex to influence the double-strand break (DSB) repair outcome (197, 203).

The technique for inserting large DNA sequences should ideally deliver efficient, programmable, and one-way insertion with desired outcomes. Several CRISPR-based homology-directed editing approaches not inducing DSBs have been developed for integrating long sequences >1 kb into genomes, thereby preserving the integrity of the host genome while achieving multi-kilobase genome recombineering. Some of these approaches include (1) the single-stranded DNA-annealing protein (SSAP) editor derived from phages, used alongside CRISPR/Cas9 or deactivated Cas9 (dCas9); and (2) the combination of recombinase/integrase with Prime Editing and dCas9. Wang et al.(2021) (204) reported REDIT (RecT Editor via Designer-Cas9-Initiated Targeting), a system that combines an SSAP called RecT with Cas9, guiding the insertion of kilobase-scale DNA sequences at specific genomic locations. They report up to 15% integration efficiencies in mammalian cell lines, including A549 and HepG2. Their efforts also highlight primary challenges with REDIT, such as the potential for off-target effects and random indel formation following DNA cutting. Wang et al. (205) also report an improved method called dCas9-SSAP Editor that uses a deactivated form of Cas9 with SSAP RecT, allowing target specificity without DNA cleavage and increased insertion efficiencies as high as 20% across various donor designs and cell types. The dCas9-SSAP editor minimises DNA damage and off-target insertions, making it highly suitable for Mammalian Display library generation and other translational applications. A method developed by Yarnall et al. (206) called PASTE (Programmable Addition via Site-Specific Targeting Elements) combines prime editing with Bxb1, achieving programmable integration of cargos up to ~36 kb with reported efficiencies in human cell lines of up to 60% and about 4-5% in primary human hepatocytes and T cells. Durrant et al. (28) identified and characterised large serine recombinases (LSRs) from microbial genomic data, expanding the toolkit for precise DNA integration in mammalian cells. Their computational approach led to the discovery of over 60 LSRs suitable for genomic engineering, with some achieving up to seven-fold higher recombination than Bxb1 and integration efficiencies of 40-75% in human cell lines such as HEK293 and K562 for payloads over 7kb. This work highlights the potential of LSRs for functional genomics applications and the integration of large DNA sequences without relying on traditional HDR approaches that require the repair of DSBs, marking a significant advancement over the previously limited efficiency and application scope of LSRs. Other techniques using λ-recombinases and LSRs for targeted transgenesis in human cells also offer new possibilities for precise genomic integration at novel loci with enhanced expression and minimal cytotoxicity (207, 208).

#### 3.1.2 Improving transgene stability

Developing cell lines that maintain transgene stability over extended periods ensures consistent expression of proteins. Recent developments in genomics, transcriptomics, gene editing and computational tools have enabled systematic identification and characterisation of multiple novel genomic safe-harbour (GSH) loci or hotspots for stable transgene integration in various mammalian cell types (155, 209–212). The continuous identification and validation of new safe-harbour loci in desired host cell lines are crucial for optimising genome editing outcomes. For instance, CRISPR/Cas9 has been employed to target safe-harbour sites such as the ROSA26 and AAVS1 in HEK293 cells, effectively allowing stable expression of therapeutic proteins over multiple generations with minimal disruption to host cell function (213). Alternative cell lines, such as the DT40 cell line derived from a Chicken B cell, have been utilised for their smaller size compared to popular cell lines such as CHO or HEK. The rapid growth of DT40 cells (doubling time ~8-10 hours vs. ~24 hours for HEK/CHO cells), and high rates of homologous recombination, are also advantageous for addressing the challenges associated with scalability of the large mammalian cell pools that are needed for the maintenance of large and diverse antibody libraries (214–217).

Mammalian systems vary greatly, and a GSH ideal for one cell type may not be effective in another. GSHs validated in human or murine cells, such as AAVS1, ROSA26 or CCR5, need to be tested in equivalent loci in the Chicken genome. Also, identification and *in vitro* evaluation of species-specific GSHs is necessary to avoid risks such as epigenetic silencing and promoter shutdown over time to achieve durable expression. Dehdilani et al. (210) used a multi-omics bioinformatics including comparative genomics and transcriptomics data to identify two new GSHs in the Chicken genome - chicken HIPP-like (cHIPP) and chicken ROSA-like (cROSA) genes yielding consistent expression of transgenes. CHO cell-based platforms are widely used for manufacturing complex therapeutic proteins, including antibodies. The development of high-producing, stable clones is essential for advancing molecules toward clinical evaluation, but this process is often time and resource-consuming due to reliance on random integration and extensive empirical screening to find the most productive clones (137). Stable integration of protein transgenes into GSHs in CHO cells can provide viable alternatives to more consistent and reliable clones (218–221). These advances in site-specific genome engineering not only enhance opportunities for antibody discovery using CHO cell-based mammalian display but also have the potential to significantly reduce timelines from discovery to Investigational New Drug (IND) application by creating stable CHO cell lines for therapeutic antibody production.

#### 3.1.3 Future directions in genome engineering

Rapid advancements in genome engineering, particularly emphasising the strategic integration of long DNA sequences, will enable next-generation mammalian display technologies. Ongoing efforts to develop more reliable integration techniques, optimise existing systems to reduce off-target effects, and reduce or avoid dependency on cell type and division state for gene editing are crucial for advancing the fidelity and efficiency of these methods (222).

### 3.2 AI/ML for predictive modelling and library design

The integration of artificial intelligence (AI) and machine learning (ML) within antibody drug development is transforming the foundational approaches to therapeutic antibody discovery and optimisation. Given the rapid evolution of AI/ML techniques, this section provides only a brief overview of their potential in predictive modelling and antibody library design for therapeutic discovery. Readers are encouraged to consult specialized reviews for a more comprehensive understanding of this topic. AI encompasses systems that can perform tasks typically requiring human intelligence, and ML enables these systems to learn and improve from experience. Deep learning (DL), a subset of ML, utilises algorithms and neural networks to analyse complex data structures, making it especially effective in handling vast arrays of biological data for predictive modelling and insights beyond traditional statistical methods. Generative models, advancing rapidly, can generate new data resembling training data, crucial for the *de novo* design of therapeutic molecules. Readers interested in deeper insights into generative models and *de novo* antibody design should refer to specialized reviews on this topic (223, 224).

Natural Language Processing (NLP) models, initially designed for text analysis, have been adapted to interpret the ‘language’ of proteins and genetic sequences, supporting the creation of synthetic antibody libraries more targeted than traditional methods relying on large physical libraries and resource-intensive screening workflows (225–227). This section explores how DL and Generative models are transforming antibody discovery through the enhanced design of antibody libraries for the optimisation of lead therapeutic candidates and how these features can potentially be used for the *de novo* creation of smaller, focused libraries for mammalian display, enhancing hit discovery early from mammalian display platforms.

Modern antibody discovery relies heavily on empirical methods like phage, yeast, or mammalian display to screen large libraries of potential candidates. These methods can be labour-intensive and iterative, sometimes requiring multiple optimisation cycles to refine antibody properties (228). In contrast, AI-driven approaches, particularly Generative models, can streamline this process by predicting and designing antibody libraries with desired specificity and affinity (229). DL and generative learning models such as variational autoencoders (VAEs) and generative adversarial networks (GANs) simulate the complex distribution of antibody features, learning from extensive protein sequences and structural data to generate diverse and novel antibody libraries (226). While these methods aim to reduce development time and resources, building reliable models capable of designing high-quality focused antibody libraries remains an aspirational goal, with ongoing research aimed at refining these predictive models.

DL models excel in predicting molecular interactions by training on diverse structural and sequence datasets. They can precisely predict how modifications to an antibody’s sequence influence its interaction with antigens, extending to modelling interactions under various physiological conditions. (230).

ML can refine library design and screening processes in mammalian display, where stable, site-specific integration of antibody variants allows early screening of highly developable antibodies (50, 56). However, due to its small library size (10^6^–10^7^) compared to phage display (10^10^–10^11^), the mammalian display is commonly used for affinity maturation and antibody engineering or to screen whole outputs from phage display in IgG format. AI-enhanced tools can be used to create more focused semi-synthetic human or humanised mammalian display libraries *de novo* with library sizes of 10^3^-10^6^ sequences optimised to exhibit optimal expression levels, stability, solubility, and low immunogenicity (225).

In the context of VHH antibodies derived from llama or other camelid sources, AI/ML have been instrumental in reducing immunogenicity and improving developability, enhancing humanization processes while maintaining high affinity and specificity (231). Techniques such as computational CDR grafting, enhanced by ML algorithms, predict the impact of modifications on binding and immunogenic profile (232, 233). BioPhi, an open-source platform for antibody design [(https://biophi.dichlab.org and https://github.com/Merck/BioPhi, date accessed: 22nd of July 2024)], automates humanisation and evaluates the humanness of sequences, representing a significant step in automating antibody library design to produce therapeutics less likely to elicit an immune response (233).

Integrating ML with high-throughput screening technologies, such as directed evolution, further underscores the utility of computational approaches in rapidly identifying and optimizing antibodies. This integration facilitates a much quicker iteration between design and validation phases, crucial for responding to rapidly evolving pathogens where the ability to quickly develop effective antibodies can significantly impact therapeutic efficacy and outbreak management (227, 234, 235).

Despite the significant advancements, challenges remain, such as the dependency on extensive, high-quality data for training predictive models. The accuracy of these models is directly tied to the breadth and depth of the training datasets, and experimental validation remains crucial to confirm their effectiveness and safety (236, 237). Additionally, the “black box” nature of many ML models can limit the understanding of underlying rules governing effective antibody-antigen interactions, necessitating more interpretable models (236, 238).

In summary, AI and ML are poised to continue their transformative impact on antibody drug development, offering innovative tools to refine the precision and efficiency of therapeutic antibody engineering using mammalian display platforms. As computational and experimental techniques become more integrated, the development of next-generation therapeutics will increasingly rely on this powerful synergy, heralding a new era of rapid, responsive drug discovery and development (236, 238, 239).

## 4 Discussion and outlook

Mammalian, phage, yeast, and ribosomal display have contributed to a progressively matured therapeutic antibody pipeline. In the continuous effort to shorten timelines and enhance the efficacy of antibody drug discovery from initial discovery to IND application, mammalian display technology presents unique opportunities. Historically, the antibody selection process has focused on affinity, but there is an increasing need to evaluate multiple properties simultaneously and prioritise developability earlier in the drug discovery process. Mammalian display allows the selection of more complex molecules in their final therapeutic format, representing an essential shift for creating stable, high-concentration formulations. Mammalian display facilitates the selection of repertoires with desirable developability properties at the library scale, supporting the development of complex antibody drugs and bi- and multi-specific biologics. Moreover, the mammalian display may have the potential to reduce timelines from discovery to IND application by selecting single stable integration antibody molecules for both discovery and high-production CHO clones. This integrated approach would address current hurdles in stable CHO cell line development and regulatory concerns about genetically defined and stable production cell lines​​.

Despite advancements in mammalian display technology, challenges remain in achieving efficient single-copy gene integration, a critical step for linking genotype to phenotype. Current approaches exhibit limitations in the high-efficiency integration of large transgenes expressing biologics such as antibodies, but future developments in genome engineering technologies may enhance this process, potentially enabling the creation of larger and more diverse libraries. Additionally, mammalian display can be combined with other technologies, such as phage display, to address the limitations of library size. Indeed, the critical need in biotherapeutic discovery is not merely generating diverse populations of binders, which can be achieved using various approaches, but efficiently identifying the best candidates for clinical development from these libraries. Mammalian display partly addresses the challenge of finding functional binders early. Mammalian display-to-secretion switching capabilities can further enhance early discovery efforts by supporting screening in the final format for desired functionality. Recent advances in high-throughput microfluidics-based single-cell screening systems have created opportunities to include biologically relevant assays in the function-first screening of mammalian secretion libraries. Additionally, the integration of AI/ML is expected to refine the discovery process by predicting binding sequences, reducing the need for large display libraries, and enabling a more focused selection of molecules in the final therapeutic format​​.

Applications of mammalian display extend beyond antibodies to a wide range of emerging therapeutic modalities, such as bispecific TCR and CAR molecules. This technology supports the development of complex biologics by selecting molecules with favourable binding and developability properties in the desired mammalian cell type (e.g., human T cell lines). There is an expanding precedent for using mammalian display systems to engineer protein classes intractable for other display technologies, such as GPCRs and proteins with complex tertiary and quaternary structures. This will expand the range of molecules to which protein engineering based on clonal selection from combinatorial libraries can be applied, potentially opening new frontiers in biologics discovery.

Overall, mammalian display technologies are poised to significantly impact the future of drug development. They offer a sophisticated platform for selecting and optimising therapeutic candidates, with potential applications extending to various classes of drug molecules, including vaccines. The ability to select for developability and functionality early in the discovery process, coupled with advancements in genetic engineering and AI/ML, positions mammalian display as a crucial technology in the evolving landscape of biotherapeutic development.
